# Using a Mobile Health Intervention (DOT Selfie) With Transfer of Social Bundle Incentives to Increase Treatment Adherence in Tuberculosis Patients in Uganda: Protocol for a Randomized Controlled Trial

**DOI:** 10.2196/18029

**Published:** 2021-01-05

**Authors:** Juliet Nabbuye Sekandi, Nicole Amara Onuoha, Esther Buregyeya, Sarah Zalwango, Patrick Evans Kaggwa, Damalie Nakkonde, Robert Kakaire, Lynn Atuyambe, Christopher C Whalen, Kevin K Dobbin

**Affiliations:** 1 Global Health Institute College of Public Health University of Georgia Athens, GA United States; 2 Department of Epidemiology and Biostatistics College of Public Health University of Georgia Athens, GA United States; 3 School of Public Health Makerere University Kampala Uganda; 4 Department of Public Health Service and Environment Kampala Capital City Authority Kampala Uganda

**Keywords:** tuberculosis, mHealth, digital health, eHealth, directly observed therapy, video observed therapy, DOT Selfie, treatment adherence, Africa

## Abstract

**Background:**

The World Health Organization’s End TB Strategy envisions a world free of tuberculosis (TB)—free of deaths, disease, and suffering due to TB—by 2035. Nonadherence reduces cure rates, prolongs infectiousness, and contributes to the emergence of multidrug-resistant TB (MDR-TB). Moreover, MDR-TB is a growing, complex, and costly problem that presents a major obstacle to TB control. Directly observed therapy (DOT) for treatment adherence monitoring is the recommended standard; however, it is challenging to implement at scale because it is labor-intensive. Mobile health interventions can facilitate remote adherence monitoring and minimize the costs and inconveniences associated with standard DOT.

**Objective:**

The study aims to evaluate the effectiveness of using video directly observed therapy (VDOT) plus incentives to improve medication adherence in TB treatment versus usual-care DOT in an African context.

**Methods:**

The DOT Selfie study is an open-label, randomized controlled trial (RCT) with 2 parallel groups, in which 144 adult patients with TB aged 18-65 years will be randomly assigned to receive the usual-care DOT monitoring or VDOT as the intervention. The intervention will consist of a smartphone app, a weekly internet subscription, translated text message reminders, and incentives for those who adhere. The participant will use a smartphone to record and send time-stamped encrypted videos showing their daily medication ingestion. This video component will directly substitute the need for daily face-to-face meetings between the health provider and patients. We hypothesize that the VDOT intervention will be more effective because it allows patients to swallow their pills anywhere, anytime. Moreover, patients will receive mobile-phone–based “social bundle” incentives to motivate adherence to continued daily submission of videos to the health system. The health providers will log into a secured computer system to verify treatment adherence, document missed doses, investigate the reasons for missed doses, and follow prespecified protocol measures to re-establish medication adherence. The primary endpoint is the adherence level as measured by the fraction of expected doses observed over the treatment period. The main secondary outcome will be time-to-treatment completion in both groups.

**Results:**

This study was funded in 2019. Enrollment began in July and is expected to be completed by November 2020. Data collection and follow-up are expected to be completed by June 2021. Results from the analyses based on the primary endpoint are expected to be submitted for publication by December 2021.

**Conclusions:**

This random control trial will be among the first to evaluate the effectiveness of VDOT within an African setting. The results will provide robust scientific evidence on the implementation and adoption of mobile health (mHealth) tools, coupled with incentives to motivate TB medication adherence. If successful, VDOT will apply to other low-income settings and a range of chronic diseases with lifelong treatment, such as HIV/AIDs.

**Trial Registration:**

ClinicalTrials.gov NCT04134689; http://clinicaltrials.gov/ct2/show/NCT04134689

**International Registered Report Identifier (IRRID):**

DERR1-10.2196/18029

## Introduction

The World Health Organization’s End TB Strategy envisions a world free of tuberculosis (TB)—free of deaths, disease, and suffering due to TB—by 2035 [1]. In 2018, TB killed an estimated 1.5 million people, with 10 million new cases of the disease worldwide [2]. Nonadherence to medication is a common, complex, and costly problem, which hampers TB control [3]. It reduces cure rates, prolongs infectiousness, and contributes to the emergence of multidrug-resistant TB (MDR-TB) strains [4-7].

MDR-TB is on the rise, with an estimated 580,000 cases reported worldwide in 2018 [2] compared to 480,000 in 2014 [8]. The World Health Organization (WHO) and the Centers for Disease Control and Prevention (CDC) recommend directly observed therapy (DOT) for TB treatment to monitor and provide treatment support for affected people whenever feasible [9,10]. When implemented properly, DOT fosters high levels of treatment adherence and early detection of adherence problems, adverse drug reactions, and worsening TB symptoms [9].

The WHO estimates that approximately 50% of patients who start treatment often fail to adhere to their prescribed medication regimens, particularly in low- and middle-income countries [11]. However, DOT is difficult to implement in low-resource settings such as Africa. Major barriers that hinder the effectiveness of DOT are TB stigma, patients forgetting to meet with TB providers or treatment supporters, the inconvenience of daily face-to-face meetings, high costs of travel, insufficient capacity of the public health workforce, and long patient waiting times at health facilities [12,13]. Therefore, innovative ways to overcome these barriers are urgently needed.

Mobile health (mHealth) tools have shown promise as alternative interventions to in-person DOT [14-16]. For example, SMS text messages and real-time electronic monitors have been used to improve adherence to antiretroviral treatment in Uganda [17]. Video directly observed therapy (VDOT) that uses a smartphone app to record videos of medication intake presents a novel way to monitor adherence remotely and overcome health system barriers of treatment delivery [18,19]. VDOT enables patients to submit videos showing daily medication intake for observation by TB care providers without the need to meet in-person. Only a few published observational studies have evaluated VDOT, including 2 from Africa [19-24]. One pilot study from Kenya showed video observed therapy to be both technically feasible and acceptable to patients and health professionals [23]. Similarly, a recent pilot done in Uganda showed VDOT to be feasible and acceptable to patients for the monitoring and support of TB treatment [24]. The efficacy of VDOT has been evaluated in 3 randomized controlled trials (RCTs) in the United States, United Kingdom, and Moldova, which have shown it to be feasible, acceptable, cost-saving, and convenient to patients [25-27]. However, to our knowledge, no published RCTs comparing VDOT to usual care have been reported in Africa.

The current study will adapt an existing VDOT platform and a smartphone app that was developed by researchers at the University of California in San Diego, California, United States [18], to suit the African context. Specifically, we will translate SMS text reminders and social bundle incentives to motivate adherence. Using mHealth tools in a context-specific way is expected to promote effective patient self-management and improve patient-provider interactions. Mobile technology has the potential to facilitate greater patient reach, anonymity, information dissemination, as well as the implementation of interventions and services in diverse contexts and settings [28,29]. Upon successful completion of this trial, we expect our findings to contribute to evidence that informs the adoption and scale-up of VDOT for use in low-income countries.

## Methods

### Trial Design

The DOT Selfie study is an open-label RCT. A total of 144 adult patients with a confirmed diagnosis of drug-susceptible TB who are initiating treatment or are within 1 month of treatment initiation at designated TB clinics will be randomized into 2 parallel groups. Of the 144 participants, 72 will be randomized into a control group to receive the usual-care DOT (UC-DOT), and 72 will be randomized into the intervention group to receive a smartphone with the VDOT app to video-record daily medication intake for review via a secure cloud system. All study participants will attend 2 monthly clinic visits at 2, 4, and 6 months (or at the end of treatment), during which clinical and sputum assessments will be performed.

### Eligibility Criteria

Participants will be included if they are (1) new or retreatment patients with clinically or microbiologically confirmed TB who initiated treatment within 1 month (we chose to enroll both new and retreatment cases since adherence barriers exist in both groups), (2) aged 18 to 65 years, (3) planning to reside in Kampala, Uganda, for the entire period of 6 months while on treatment to facilitate close follow-up, (4) able to provide signed informed consent, and (5) able to speak and read English or Luganda (the local dialect). Participants will be excluded if they (1) are confirmed to have drug-resistant TB (MDR- or XDR-TB); (2) are very ill patients (ie, those who feel that they would not be able to withstand 2 hours of study procedures at enrollment; (3) have a cognitive, motor, visual, or hearing disability that prevents full participation in VDOT (eg, disabilities that prevent holding a phone or an inability to swallow medication as whole pills); and (4) do not have access to a power source to charge the smartphone or reside in areas without cellular network coverage.

### Study Setting and Recruitment

**Figure 1:**
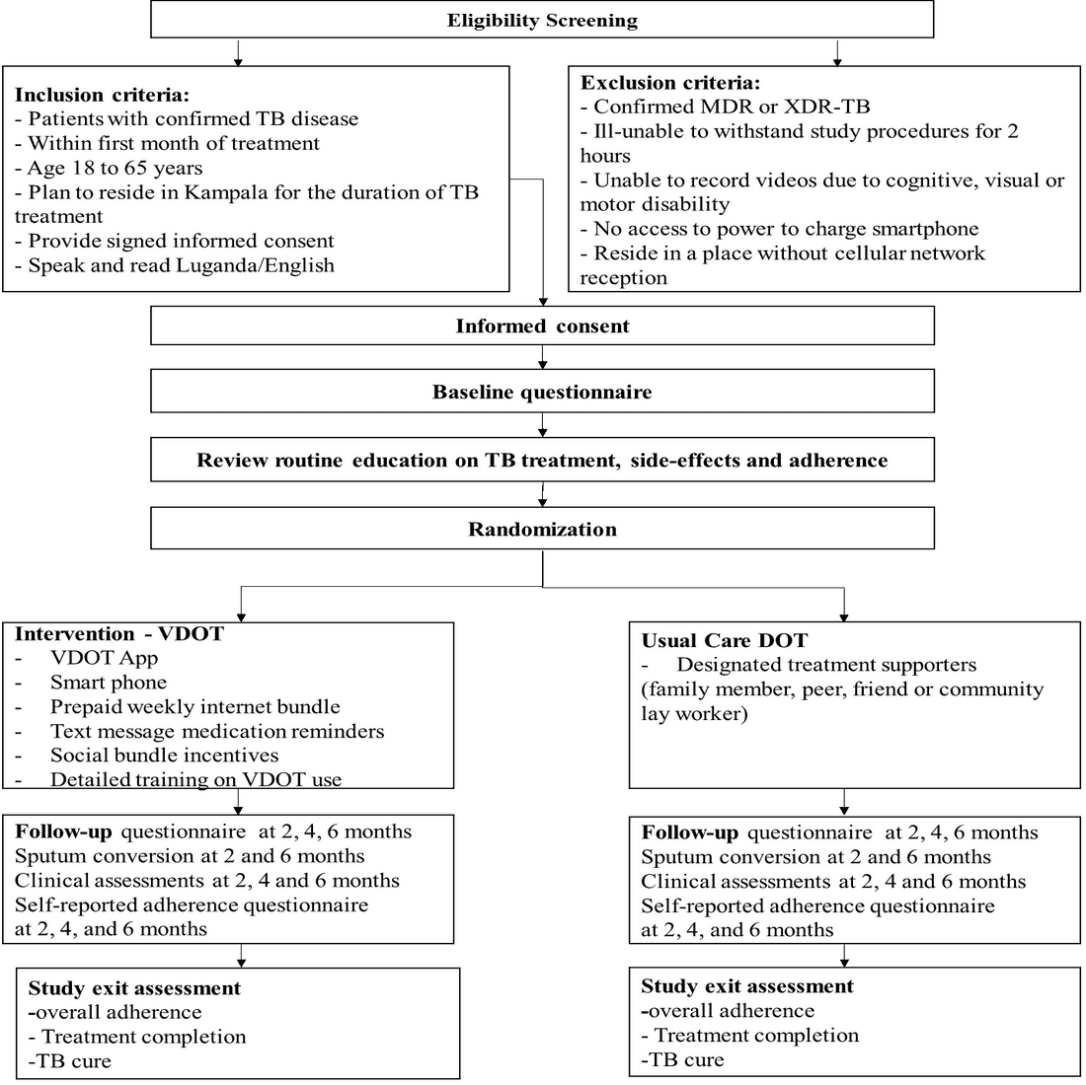
DOT Selfie participant flow diagram. DOT: directly observed therapy; MDR-TB: multidrug-resistant tuberculosis; TB: tuberculosis; VDOT: video directly observed therapy; XDR-TB: extremely drug-resistant tuberculosis.

The primary study site will be the Lubaga TB clinic in Kampala, Uganda. Other public clinics, such as Kawala, Kitebi, or Kisenyi, which provide free TB services, will be used as secondary sites to supplement study recruitment. The study clinic sites are all located about 10-15 km from Kampala city center and, together, treat approximately 1000 TB patients annually. We estimate that a total of 8-10 patients per week, or 32-40 patients per month, will be enrolled by the research team. Since the selected sites treat roughly 90-100 TB patients a month, we will use consecutive sample selection to enroll every eligible subject in each arm until the required sample size is achieved. With this recruitment rate, we expect to complete participant enrollment within 4-5 months. The primary recruitment strategy is to screen patients who are newly diagnosed or those that will visit the clinic within the first month of their treatment. Eligibility will be assessed by a trained research assistant who will describe the study to the eligible patient and answer any study-related questions. Patients who are interested in participating will be required to provide written consent, after which they will be invited to complete a baseline questionnaire (Multimedia Appendix 1). The baseline questionnaire will ask questions about (1) patients’ TB diagnosis and initial treatment; (2) socio-demographics and income; (3) experience with cellphones, smartphones, and technology; (4) transportation and other costs; (5) social and personal life; (6) knowledge of TB disease and treatment; (7) privacy and confidentiality concerns; (8) current family and friend support; and (9) perceived stigma from the community. All participants will be educated on TB, including treatment, side effects, and the need for adherence. Thereafter, they will be randomized to one of 2 groups. The participant flow is described in Figure 1.

### Study Procedures

#### Randomization

We will randomize 144 participants with an equal allocation ratio of 1:1 to receive either VDOT or UC-DOT. An equal allocation was chosen to optimize the power of the study [30]. Permuted block randomization with block sizes of 4 and 6 will be used to allocate participants to intervention or control groups while maintaining balance across groups. Each block will have a specified number of randomly ordered treatment assignments [30]. Additionally, stratified randomization will be done according to sex to ensure equal representation of men and women in each study group [30]. The stratification is justified because sex is an important variable that influences adherence to TB treatment [31]. A computer program will randomly generate study arm assignments within block sizes of 4 and 6. This randomization will minimize the likelihood that the study team will be able to predict the next study arm assignment. Each block will contain equal proportions of control and intervention. Within each block will be 4 or 6 sealed, opaque allocation envelopes, depending on the block size. Blocks and allocation envelopes will be sequentially numbered according to the computer-generated randomization schedule. Each allocation envelope will contain an assignment card on which the study group assignment (UC-DOT or VDOT) will be printed. To assign a patient to a study group, blocks will be selected in their sequential order. Allocation envelopes within them will be selected sequentially as well. Each envelope will be opened only after the participant details have been written on it. Carbon paper inside the envelope will transfer participants’ details to the assignment card. Allocation envelopes will be sealed using tamper-proof security tape.

#### Blinding

Due to the nature of the interventions, it is not possible to blind study participants or research nurses and interviewers after assignment to a study arm. This is because, as part of the study procedures research, nurses must observe participants swallow their pills via VDOT, and at follow-up visits, interviewers must collect participant data regarding medication adherence. The research staff allocating participants to study groups during randomization will be blinded to the sequence. This is to minimize the likelihood that the research staff will be able to predict the next study arm assignment. Study investigators will be blinded to the allocation and any preliminary analyses before the end of the study. Only the trial statistician will be unblinded to the analyses. The data collection tools used for this purpose include information about how pill-swallowing is conducted (UC-DOT or VDOT), thus making the study group assignment obvious.

#### Description of Standard Procedures

The trial and intervention are described according to the SPIRIT (Standard Protocol Items: Recommendations for Interventional Trials) guidelines [32]. Regardless of the assigned study group, all participants will attend an initial face-to-face educational session, which will provide information on (1) the importance of medication adherence according to the daily TB drug regimen; (2) the correct way to take TB pills, including dose, timing, and the importance of taking pills whole; (3) storage of pills and what to do in the event of a missed dose; and (4) the importance of reporting (in-person or through the VDOT app) any problems related to TB medications, such as side effects, adverse events, symptoms, lost pills, lost or malfunctioning smartphone or app, etc, as well as any study-related issues.

#### Control: Usual-Care Directly Observed Therapy (UC-DOT)

Participants will receive UC- DOT as administered in routine clinical practice under the Uganda National TB program. Routine DOT typically involves treatment observation 3-5 times per week by a designated treatment supporter who could be a trained community worker, a volunteer lay-worker, a family member, a friend, or community linkage facilitator, with the weekend doses self-administered. The name and phone contact details of each designated treatment supporter for each patient are documented. The treatment observation and support occur at any location mutually agreed upon between the patient and their treatment supporter. To document adherence, the patient records daily doses taken on a predesigned treatment card issued at the clinic and returns it at every routine visit. Adherence is further assessed using patient self-reports, pill counts, and clinic attendance for prescription refills. Study research assistants will work in partnership with designated treatment supporters to collect study information on adherence from clinic records coupled with a structured adherence questionnaire at follow-up visits. Patients in the UC-DOT control group will be followed up for missed doses and routine visits according to the National TB program guidelines. Research staff will only follow up with participants for scheduled study visits and procedures.

#### Intervention: Asynchronous Video Directly Observed Therapy (VDOT)

**Figure 2:**
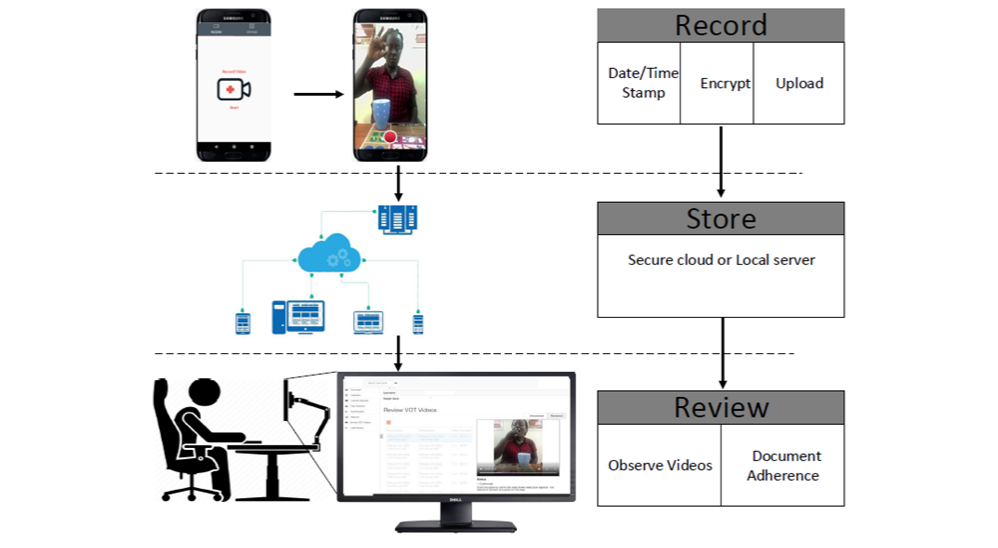
Schematic of asynchronous video directly observed therapy (VDOT) system.

Upon assignment to the intervention study group, participants will receive detailed training (in English or Luganda) on the VDOT app using a training manual provided by the VDOT software developer. The asynchronous VDOT intervention comprises a smartphone with a SIM card, the preloaded VDOT app, a free weekly internet data subscription of 350 MBs paid for by the study at commercial rates, and daily SMS text message medication reminders. The VDOT app is a free downloadable app through the App Store, and the system is Health Insurance Portability and Accountability Act (HIPAA)-compliant and has been validated [33]. Additionally, patients will receive a weekly incentive in the form of social bundles or airtime minutes each time they successfully submit videos for 7 consecutive days. The smartphone will have a unique phone number to ensure that SMS reminders and other phone communications are sent specifically to study participants. The VDOT app is free and downloadable from the Play Store. A unique personal identification number (PIN) will be assigned to each person at registration in the VDOT system to facilitate login into the VDOT app. The PIN will be a security feature that will prevent nonstudy participants, such as other family members or friends, from accessing the app. The app is equipped with video recording features that allow patients to record videos of themselves swallowing pills 7 days each week (Figure 2). To ensure safety and to address concerns that side effects may be missed with limited face-to-face contact, patients are also encouraged to report any adverse events on the daily videos. After the recording ends, an automatic, encrypted, time-stamped video is transferred through a cellular connection and uploaded to a secure cloud server for storage and playback. This enables patients to securely and confidentially record and submit daily medication doses. Once a submission is completed, the patient cannot retrieve or access the video. The smartphone will automatically receive the internet bundles to facilitate video submission.

**Figure 3:**
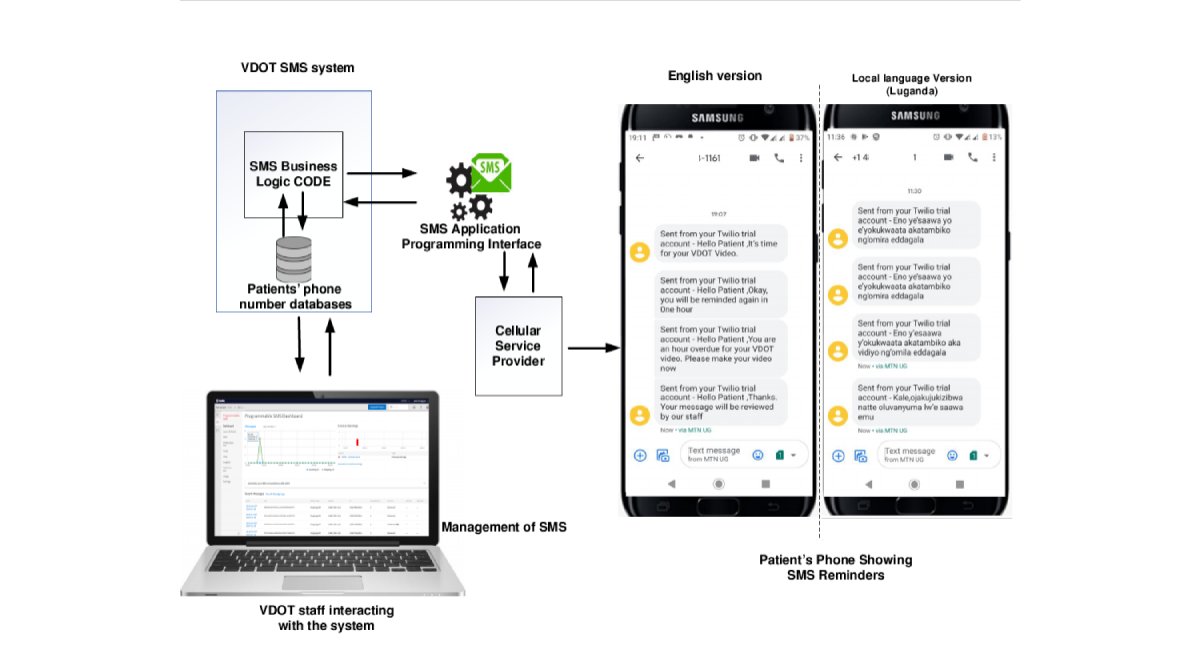
Video directly observed therapy (VDOT) SMS text messaging system.

The VDOT system has a feature to adapt the text messages either in English or Luganda (Figure 3). These text messages will include reminders to motivate patients to continue taking their medications. An example of these messages is “It’s time to take your pills and send a video. Taking your pills will help you get better.” A single SMS text will be sent to each participant in the morning as a reminder; a second text will be sent within 8 hours of the first if a video is not received by the system.

At the clinic, a trained study nurse will log into a secured, HIPAA-compliant VDOT system via a tablet or laptop to download and review patients’ daily videos and document medication adherence. On days when video recording or uploads are not possible, medication adherence will be assessed using patient self-reports, pill counts, and prescription refills at the clinic. The nurse will thus be able to track missed doses and reported side effects, and follow up with appropriate support or advice. Participants will sign an agreement to return the smartphone to the clinic upon completing their full course of treatment.

#### Follow-up of Participants With Missed Videos or Study Visits

A predefined follow-up protocol will be used to contact study participants in cases of missed doses or videos. The research staff will make 2 phone call attempts within the first 24 hours of a missed, expected video to establish dosing history and the reason for missing videos. If there is no response from the participants within 72 hours, the research team will escalate the follow-up to a field visit to trace the participant at home or work. After a period of 2 weeks from when the intensive, active follow-up protocol has been completed, there will be a waiting interval of 2 weeks, hoping that the participant will call back or return to the clinic for a routine visit. If the participant does not show up for the scheduled clinic visit, the national TB program staff will get involved in support of the patient. A declaration of “lost to follow” will be made if the participant does not return for 2 consecutive months, in line with the standard World Health Organization’s guidelines [34].

#### Criteria for Discontinuing or Modifying Allocated Interventions for Trial Participants

Patients may voluntarily withdraw from the study for any reason, at any time. After consultation with the protocol chair, the investigator may also withdraw participants from the study to protect their safety or if patients are unwilling or unable to comply with the required study procedures. Reasons for all cases of nonadherence and nonretention will be retrieved and documented in case report forms. Randomized patients who are prematurely discontinued from the study at any time will have their clinical and laboratory evaluations performed, if possible, and will also continue receiving treatment for TB and routine health services. According to standard definitions provided by Friedman et al [35], a participant who is assigned to the VDOT but who fails to adhere to the intervention will be considered a dropout, whereas a participant who discontinues the intervention but continues to take treatment under the usual-care procedures will be considered a cross-over [35]. Participants who withdraw, dropout, or cross-over will be included in the primary intention-to-treat analysis.

### Study Outcomes

The primary outcome measure is the level of adherence calculated as the fraction (proportion) of expected doses observed (FEDO) over the 6 months of treatment or by the end of the study following randomization. Since direct observation as a way of measuring medication adherence may be challenging to employ consistently in the UC-DOT group, we will also use self-reports and pill counts as indirect measures of adherence and then triangulate the results. Time-to-treatment completion will be assessed as the main secondary outcome. Other outcomes will be sputum smear conversion at 2 and 6 months or the end of treatment, clinical response as a measure of self-reported improvement in TB symptoms, and weight gain at 2, 4, and 6 months or the end of treatment. Other outcomes will be self-reported side effects, the occurrence of adverse events, and patient satisfaction at the exit of the study. Patient satisfaction will be evaluated in 2 ways: (1) overall satisfaction with VDOT or UC-DOT, and (2) satisfaction with specific areas, such as the VDOT technology and app use; information and training received; interaction with treatment supporters, health providers, or research staff; and privacy and confidentiality during medication intake. Additionally, at study exit, we will conduct individual qualitative interviews for both UC-DOT and VDOT participants to capture detailed information about participant experiences during the study.

Study measurements will be done at baseline and follow-up at months 2, 4, and 6 to ensure close monitoring of all patients in treatment. All participants, regardless of the study group, will complete a questionnaire at these follow-up visits (Multimedia Appendix 2 and 3). The follow-up questionnaire will address issues such as (1) patients’ current use and any changes in experience with a cell phone, such as acquiring a personal cell phone or smartphone; (2) transportation and other costs to and from the TB clinic; (3) TB treatment experiences using VDOT or UC-DOT; (4) experiences when using a phone to record videos or when receiving support under the usual DOT; (5) satisfaction with TB and VDOT or UC-DOT; (6) ongoing support received during treatment; (7) side effects experienced; (8) difficulties with medication supply or transportation to the clinic; and (9) privacy, confidentiality, and stigma concerns. For internal validity, adherence will also be measured at follow-up visits by pill counts (when possible) and using a 10-item self-reported adherence questionnaire [36] (Multimedia Appendix 4).

### Qualitative Exit Interview

In-depth interviews will be conducted at the exit of the study to gather detailed information to enrich the understanding of the quantitative data. This will involve intensive individual interviews with a small number of study participants to explore their general experiences, operations, processes, and outcomes that they perceive as resulting from their involvement in VDOT or usual-care DOT. We will also specifically probe the participants’ experiences of stigma, privacy, gender, and socio-cultural issues. To ensure representation of a wide variety of views, participants will be purposively selected based on sex, age, and observed experiences during the time in the study. For example, we will ensure both younger and older and male and female participants are included. In terms of treatment experiences, patients with low, moderate, and high adherence will also be selected accordingly. We will aim to interview a total of 20-30 patients with equal numbers from each study arm. The final number of participants to be interviewed will be determined based on the attainment of thematic saturation (ie, the point at which interviewing more respondents will yield no new information on the topics of interest) [37]. Interviews will be audio-recorded and transcribed. We will use thematic analysis to code, identify key patterns or themes, and then summarize the findings. ATLAS.ti software (version 9; ATLAS.ti Scientific Software Development GmbH) will be used in the coding and detailed processing of the data.

### Data Management

All study questionnaires will be administered using electronic forms on ODK (Open Data Kit) using laptops or tablet devices. Data in the form of videos collected from the VDOT system will be held on a secure HIPAA-compliant cloud server and will be backed up nightly locally in Uganda. Weekly data integrity reports will be run on the system using an automated routine. Monthly data exports will be made, and data will be merged into the master data file. The master data file will be stored at the University of Georgia on a password-protected network with access restricted to study personnel. This will be automatically backed up daily. The master data file will use the unique study number allocated at the time of randomization but will not include personal identifier information. This identifiable information will be held in a separate password-protected file on the same network drive in the form of a lookup table.

### Sample Size

This trial is powered on the primary outcome measure and based on a comparison of the medication adherence level between the UC-DOT group and the VDOT group. The calculated sample size is 124 in order to achieve a chi-square test difference of 0.85 versus 0.63 (difference 0.22 and odds ratio 0.30) between the groups. We assumed an attrition rate of 14%, guided by published literature; this was based on a randomized controlled trial comparing a digital adherence intervention and usual care in Kenya in which the overall loss to follow-up rate was 11.7% (9.9% in the usual-care DOT arm and 1.76% in the digital intervention arm) [38]. By inflating the attrition rate to 14%, we erred on the higher side of attrition. In contrast, another RCT of asynchronous VDOT done in the United Kingdom had a higher attrition rate of 23%; however, the study population was very different from our current study in that it included 58% of homeless subjects [15]. At a 14% attrition rate, the estimated final sample size was 144 participants with 72 subjects per group. This would provide a power of 80% to detect a 22% difference in the primary outcome between the 2 comparison groups (85% VDOT vs 63% DOT) based on a 2-sided significance level of 5%. Sample size tables were used to estimate precision. All calculations were performed using the SAS statistical software package (version 9.3; SAS Institute).

### Statistical Analyses

Intention-to-treat analysis will be conducted where all patients will be analyzed according to the arm to which they were originally randomized. VDOT treatment observations will be classified as completed if ingestion of all medicines is observed, or if videos are received but not viewable because of a technical complication (given that patients would have no control over whether videos were corrupted). The sensitivity analysis will consider only videos for which all medications are observed as completed. An adherence proportion will be observed for each individual in this study. Let p_ij_ be the adherence proportion for individual j (j=1, ..., n) in treatment group i (i=0 for VDOT and i=1 for UC-DOT). Then, let p_0_ and p_1_ equal to the average FEDO in the VDOT and UC-DOT arms, respectively. Similarly, let s_0_=s.e.( p_0_) be the standard error of p_0,_ and similarly, let s_1_ be the standard error of p_1_. The standard *t* test statistic will be used to calculate a *P* value based on the following statistic:

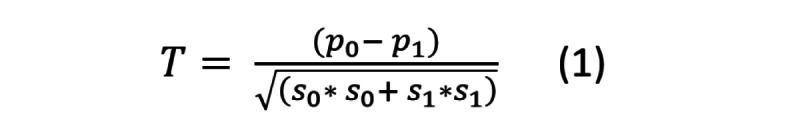



The Welch *t* test will be used to calculate significance. However, since we do not have estimates of s_0_ and s_1_, this formula is not useful for power calculation. Therefore, to calculate power and sample size, we treat the response for each individual as binary (0 or 1), indicating adherence or nonadherence. This corresponds to using an adherence cutoff value of FEDO with equal-to or greater than 80% as a threshold for adherence. This binary response variable for adherence will be used in the final analysis to facilitate comparison with previous clinical trials [15].

Univariate statistics will be used to describe the baseline characteristics of the study population, the prevalence of individual clinical responses, and the level of adherence. Similar analyses will be repeated for secondary outcomes. Multivariable logistic regression analyses will be done to test for significant associations between study groups (VDOT/ UC-DOT) as the main exposure and the primary binary outcome of adherence (≥80%; yes/no). Age and HIV status are set a priori as potential confounders that will be adjusted for in all the models. Sex will not be adjusted since the randomization is stratified by sex. Other covariates that will be adjusted for in the models include education level, income, and smartphone ownership, previous TB treatment, HIV status, and other clinical variables at baseline. Crude and adjusted odds ratios with 95% confidence intervals will be presented, and statistically significant *P* values will be less than .05.

Secondary analyses of time-to-treatment completion will provide another outcome that can be used to compare the 2 groups according to TB program performance indicators. Survival analysis to compare the median time-to-treatment completion between study groups with associated log-rank tests will be performed. Crude and adjusted Cox proportional hazards regression analyses will be performed to determine factors associated with time-to-treatment completion. Cox proportional hazards ratios with corresponding 95% confidence intervals will be presented. Other analyses comparing trends at months 2, 4, and 6 in adherence, sputum conversion, and clinical response will be performed. These repeated measures data will be modeled as a discrete-time Markov chain or Poisson process [30]. Analyses will be done using Stata (version 14; StataCorp) and R (version 3.3.2; R Core Team) software.

### Plan for Handling Missing Data

We expect that study participants may drop out or miss visits, thus producing missing data and compromising the ability to conduct an intent-to-treat analysis and draw conclusions about the causal link between the method of monitoring and medication adherence. Since there is no universal method of analyzing missing data [39,40], we will follow the guidelines of the National Research Council (NRC) on the handling of missing data in clinical trials [41]. We will focus on 2 critical elements: (1) careful conduct of the study to limit the amount of missing data, and (2) analysis that makes full use of information on all randomized participants. Careful attention will be paid to assumptions about the nature of the missing data underlying estimates of treatment effects. We will use approaches identified by Little and Rubin [42,43] based on 3 categories for classifying how missing data are generated: missing completely at random (MCAR), missing at random (MAR), and missing not at random (MNAR).

### Data and Safety Monitoring

Since this pilot RCT study employs a low-risk intervention, is not blinded, and does not involve vulnerable populations, we plan to perform a limited scope of data and safety monitoring. The main focus will be on the safe execution of the study protocol as planned [44]. The principal investigators and co-investigators of the study have constituted an independent data monitoring committee to ensure that the trial is conducted according to the approved protocol, including participant recruitment, accrual and retention, participant risk versus benefit, adverse events, periodic assessments of data quality, timeliness, and other factors that may affect study outcomes. All adverse events will be reported to the University of Georgia Institutional Review Board and the Makerere School of Public Health Research Ethics Committee. Summary reports of adverse events will be made to the National Institutes of Health (NIH) in the progress report at the end of month 4, 6, and the final report, unless the nature of a particular event warrants reporting to NIH immediately.

Interim monitoring will be performed after enrolling 50% (36/72) of patients in the VDOT intervention and control groups. This will be done to ensure that the study is conducted according to the protocol and also to check on the level of adherence in both study groups. All analyses will be led by the study biostatistician. Stopping rules will not be applied to this pilot RCT because there is no evidence to inform the threshold for the clinically important difference (ie, the minimum magnitude of the treatment benefit large enough to offset the treatment harms) [44,45]. Stopping the trial would be justified only on the basis of strong evidence of net benefit or harm [45], conditions which are rarely met in small pilot trials like our study.

### Plans for Collecting, Assessing, Reporting, and Managing Solicited and Spontaneously Reported Adverse Events and Other Unintended Effects of Trial Interventions or Trial Conduct

An adverse event in this study is any untoward medical occurrence in a patient that is temporally related to the research, whether or not it is related to the intervention or the patient's participation in the research. Data on all adverse events will be collected after informed consent has been provided and patients have been enrolled in the study. This will continue throughout the study. If a patient experiences an adverse event after providing informed consent but has not started to receive the study intervention, the event will be reported as not related to the study intervention. An adverse event that meets the criteria for a serious adverse event between study enrolment and the end of the study will be reported to the local institutional review board as a serious adverse event.

A serious adverse event for this study is any untoward medical occurrence that is believed by the investigators to be causally related to the study drug and either results in death, is life-threatening, results in severe or permanent disability, requires inpatient hospitalization, or is a significant hazard as determined by the data monitoring committee. Serious adverse events that occur after a patient is discontinued from the study will not be reported unless the investigators believe the event may have been caused by a study intervention or protocol procedure. This will be determined based on a temporal relationship to the study intervention and whether the event is unexpected or unexplained given the patient’s clinical course, previous medical conditions, and concomitant medications.

Potential serious adverse events will include lost to follow-up, death from tuberculosis, data security breaches, violence toward study personnel during patient interaction, complaints about the study from patients, or study clinics. These will be reported to the study coordinator and the study chief investigator, who will look into the matter and discuss it with the affected patient’s case managers, and findings will be reported to the trial steering committee and data monitoring committee.

## Results

The study was funded in July 2019 and has been approved by the institutional review boards of the University of Georgia (November 12, 2019) and Makerere University (February 26, 2020). Due to the COVID-19 pandemic, study enrollment was delayed for 4 months. Participant enrollment into the RCT began in July and will continue through November 2020. Follow-up will continue through June 2021, when data collection is expected to be completed. Analysis, interpretation, and preliminary dissemination of results are planned for June 2021 to August 2021, through local workshops and scientific conferences. The main results are expected to be published by December 2021. Since the study is being conducted during the ongoing COVD-19 pandemic, there might be unintended negative impacts on the delivery of usual care with DOT. Access to treatment for people with TB is likely to be interrupted as community health workers, doctors, and laboratories devote their energies and resources to the COVID-19 response. There is a potential risk that prevention and treatment programs for the existing conditions will be disrupted. Although such disruptions could inflate the effectiveness of VDOT given that the technology use limits in-person contact, we can only speculate on the magnitude of this effect. The degree to which study findings will be generalizable to nonpandemic times may also be altered. To address some of these concerns, questions related to the impact of COVID-19 on access to routine TB services have been included in the baseline and follow-up questionnaires. This will facilitate further interpretation of the results in the context of prevailing circumstances. Separate studies are needed to evaluate the impact of COVID-19 on the health system and patient care.

## Discussion

### Rationale

Nonadherence to treatment in patients with TB is a serious obstacle to the realization of the World Health Organization’s End TB Strategy goals [46] because it is responsible for the emergence of multidrug resistance and prolonged periods of infectiousness [4,47]*.* Currently, there is a lack of universally feasible, acceptable, and effective strategies to monitor and support adherence to TB treatment. This creates a critical need to evaluate VDOT applications under more diverse conditions and settings to define their function and compare them with traditional approaches to treatment monitoring [48,49]. Mounting evidence suggests increased utility and effectiveness of accessible mobile technologies in enhancing the monitoring of patients with chronic diseases [48-50]. The increased utility can be attributed to the increasing affordability and reliability of smartphones in both high- and low-income settings, while the expansion of cellular and internet networks in developing countries is responsible for the effectiveness of mHealth technologies. Therefore, VDOT is a promising alternative way to mitigate the adverse impact of nonadherence on individuals, families, and communities, especially in underserved populations in Africa.

### Ethical Implications

As it is currently practiced in Uganda, in-person DOT has the potential to be stigmatizing for patients, as it may result in unwanted public disclosure of patient disease status. This fear of—or reality of—stigmatization can negatively impact patient autonomy in regard to treatment-seeking behavior and treatment adherence [51]. However, mHealth technologies such as VDOT are likely to be less stigmatizing as they do not require health workers to visit patients at home or work. The remote nature of interaction minimizes the chances of unintended exposure of private information. The flexibility of making video recordings at the patient’s desired time and place greatly enhances privacy [46]. Patients agreed that VDOT promotes autonomy and a sense of control over their health [18]. Nonetheless, even with the use of VDOT, the potential for stigma, unintended disclosure, and a loss of autonomy could still be present given that videos contain identifiable information like the patients’ faces. At the study exit, we will conduct qualitative interviews with study participants selected to discuss their varying experiences with both VDOT and UC-DOT successes, concerns, and failures that may have affected their adherence to treatment during the study.

This protocol describes the design and methods of a randomized control trial assessing the effectiveness of using VDOT plus incentives to improve medication adherence in TB treatment in Uganda. This RCT leverages the accessible features of mobile phone technology to deliver VDOT remotely, in a less intrusive and less cumbersome way, than in-person DOT. It explores a patient-led management approach that is more convenient, enabling patients to take medications on their schedule.

### Strengths in the Context of Prior Work

The proposed study is innovative because it is the first time that an RCT will be conducted to evaluate VDOT in an African setting. The control participants receiving usual-care DOT will provide an important comparison that will enrich our understanding and inform future interventions using VDOT and other mHealth programs. Fundamentally, Kampala, Uganda (the proposed site of the pilot study), has a significantly higher incidence of TB disease (201 cases per 100,000 in 2017) than any of the other locations where VDOT has been evaluated to date [2].

### Potential Limitations

We anticipate that a few smartphones may be lost or stolen throughout the study duration. This study will document any phone losses and replace phones to allow participants to continue in the study. Phones may run out of battery power and turn off during video recording. To minimize this issue, patients will be trained and encouraged to ensure that phones are always charged nightly or before recording videos. Interruptions in the electrical power supply may prevent patients from charging their phones to take videos when taking their medications. As such, some adherence records may be incomplete; information gaps will be filled by using alternate data sources such as self-reports [36]. Cellular network connectivity problems may limit VDOT recordings by patients; unless patients travel to remote, rural areas of Uganda, we expect this situation to be rare. Although pill counts and self-reports are indirect measures of adherence, they are acceptable proxies in the absence of more direct demonstrations of adherence [36]. As described above, VDOT has less potential to infringe upon patient autonomy and cause stigmatization compared to the in-person DOT. Lastly, the results of the study will be generalizable to TB patient populations in urban settings in the context of the ongoing COVID-19 pandemic. However, infrastructural and technical limitations may prevail against the implementation of VDOT or similar technology interventions in rural settings.

### Conclusion

The significance of this study is at least 3-fold. First, the expected positive impact is the potential to adapt and scale a technology-based alternative to the DOT strategy, one that is contextualized to the local setting, convenient, and cost-saving for patients and the health care system. This RCT is highly relevant because the setting (Kampala, Uganda) has a much higher incidence of TB than any of the other locations in which VDOT has been evaluated to date. Second, it will provide the first RCT data on the use of VDOT in an African country. According to the NIH, “there is a need to stimulate research utilizing mHealth tools aimed at the improvement of adherence to treatment, effective patient-provider communication, and self-management of chronic diseases in underserved populations” [52]. Third, this pilot RCT will be a first step to meeting the urgent need to develop new mHealth tools and test existing ones for contextual acceptability, feasibility, and efficacy to mitigate the serious public health consequences of medication nonadherence. Our long-term goal is to revolutionize patient monitoring, improve patient-provider communication, and promote self-management by utilizing mobile health tools that are contextualized to the African setting.

## Appendix

Multimedia Appendix 1 Baseline questionnaire, version 2.1.

Multimedia Appendix 2 Usual-care directly observed therapy (UC-DOT) follow-up questionnaire.

Multimedia Appendix 3 Video directly observed therapy (VDOT) follow-up questionnaire.

Multimedia Appendix 4 Self-reported adherence questionnaire.

Multimedia Appendix 5 NIH peer-review report.

## Abbreviations

DOT: directly observed therapy
FEDO: fraction of expected doses observed
HIPAA: Health Insurance Portability and Accountability Act
MDR-TB: multidrug-resistant tuberculosis
mHealth: mobile health
NIH: National Institutes of Health
PIN: personal identification number
RCT: randomized controlled trial
TB: tuberculosis
UC-DOT: usual-care directly observed therapy
VDOT: video directly observed therapy
XDR-TB: extremely drug-resistant tuberculosis
