# Supplementary material for: Using a Mobile Health Intervention (DOT Selfie) With Transfer of Social Bundle Incentives to Increase Treatment Adherence in Tuberculosis Patients in Uganda: Protocol for a Randomized Controlled Trial
Source: JMIR Res Protoc. 2021 Jan 5;10(1):e18029. doi: 10.2196/18029 (PMC7815451; doi:10.2196/18029)
Supplement: Multimedia Appendix 1 [file resprot_v10i1e18029_app1.pdf]

BASELINE VDOT SURVEY QUESTIONNAIRE 2.1

section A: Participant Information

Thank you for your voluntary participation in the DOT Selfie study. My name is [NAME of Interviewer], and I work for the Makerere University and University of Georgia in partnership with the National TB program. As you now know, we are evaluating ways of monitoring and supporting patients while they take their TB medications by using either mobile phone videos or a health worker or treatment supporter watching a person face-to-face. In this initial interview we would like to ask some questions about yourself, experience with cellphone, your treatment and personal lifestyle. Do you have any questions? Okay, let's begin

Q1. Enter Height

in cm

____

Q2. Enter weight

in Kg

____

Participant Information

Q3. Please Enter 5-Digit Participant Study ID

____

Q4. Record NTLP registration

please copy as it is on the card

____

Q5. Enter study assigned phone number

enter 10 -digit number e.g 0701010101

____

Q6. Please Select Site of enrolment and Interview

- ◯ Lubaga clinic
- ◯ Kitebi clinic
- ◯ Kawaala Clinic
- ◯ Other

please specify

____

Q7. Where does the participant reside?

- ◯ Lubaga division
- ◯ Kawempe division
- ◯ Nakawa division
- ◯ Makindye division
- ◯ Kampala Central division
- ◯ Other

specify

____

section B: General and sociodemographic information.

The first set of questions will be about your general information, education and socioeconomic background.

Q8. What is your sex?

- ◯ Male
- ◯ Female

Q9. How old are you?

Age in complete years

____

Q10. What is the highest level you completed in school?

- ◯ No formal education
- ◯ Primary 1-7
- ◯ Senior 1-4
- ◯ Senior 5-6
- ◯ Certificate/Diploma
- ◯ Tertiary/University

Q11. What is your religion?

select one

- ◯ Catholic
- ◯ Protestant
- ◯ Muslim
- ◯ Pentecostal
- ◯ SDA
- ◯ Other

please specify

____

Q12. What is your marital status?

- ◯ Single/never married
- ◯ Married/cohabiting
- ◯ Separated/divorced
- ◯ Widow/widower

Q13. Are you currently employed?

- ◯ Yes
- ◯ No

Q14. Do you currently work for pay?

- ◯ Yes
- ◯ No

Q15. What type of employment do you have?
select all that apply

- ☐ Employed full time away from home
- ☐ Employed part time away from home
- ☐ Work to earn income at home
- ☐ Odd jobs, temporary work
- ☐ Own business in town
- ☐ None
- ☐ Other

please specify

____

Q16. How much would you estimate as your average total personal income in a typical month from all sources including jobs, businesses, and support from family or friends?
UGX currency

____

Q17. How many people live in your household including yourself, children and adults?

____

Q18. How many of your household members are...?

- ◯ Less than 18 years old only ?
- ◯ 18 years old and above?
- ◯ Both less than 18 & above 18 years
- ◯ No household member

enter number of members less than 18 years

____

Enter a number of members above 18 years

____

enter number of members less than 18

____

enter number of members greater than 18

____

Q19. How much would you estimate as your average total household income from all household members who work in a typical month from all sources including jobs, businesses, and support from family or friends?

- ◯ UGX
- ◯ Don't Know

Enter amount

____

Section C: Participant TB diagnosis and Treatment History

Participant TB Diagnosis and Treatment History

The next set of questions are about TB diagnosis and initial treatment. Please remember that your answers will be kept confidential. The information we gather from this interview will not be used to identify you individually.

Q20. Have you ever been treated for TB before this episode?

- ◯ Yes
- ◯ No

Q21. What was your TB diagnosis based on

- ◯ Sputum Microscopy
- ◯ GeneXpert MTB/RIF test
- ◯ Clinical symptoms
- ◯ Chest X-ray
- ◯ Not done

please specify

- ◯ Smear grade 3+
- ◯ Smear grade 2+
- ◯ Smear grade 1+
- ◯ Smear grade scanty

please specify

- ◯ Low
- ◯ Medium
- ◯ High

Enter chest X-ray results at baseline

- ◯ Normal
- ◯ Abnormal

Q22a. Did you develop a cough at the start of your TB disease?

- ◯ Yes
- ◯ No

Q22b How would you describe your cough?

- ◯ Mild
- ◯ Moderate
- ◯ Severe

Q22c. Was your cough accompanied by sputum production?

- ◯ Yes
- ◯ No

Q22d. How would you describe the sputum?

- ◯ Scanty and non-bloody
- ◯ Copious and non-bloody
- ◯ Scanty and bloody
- ◯ Copious and bloody

Q22e. What other symptoms did you have?

select all that apply

- ☐ chest pain
- ☐ weight loss
- ☐ loss of appetite
- ☐ Fever
- ☐ difficulty in breathing
- ☐ excessive night sweating
- ☐ None
- ☐ Others

Q23a. For how long did you cough before you received a diagnosis of tuberculosis?

- ○ days
- ○ weeks
- ○ months

Enter number of days

____

Enter number of weeks

____

Enter number of months

____

Q23b. How would you rate your overall health currently?

- ○ Excellent
- ○ good
- ○ poor
- ○ very poor

Q24a Are you currently on TB treatment?

- ○ Yes
- ○ No

Q24b. For how long have you been on TB treatment?

- ○ Less or equal to one month
- ○ Greater than one month

24c.Do you have a designated treatment supporter? (This is a person you have registered at the clinic to support you as you take your TB medications).

- ○ Yes
- ○ No

Q25. Did any member of your household ever receive treatment for TB?

- ○ Yes
- ○ No

section D: Participant experience with cellphone, smartphones and technology

Participant experience with cellphone, smartphones and technology

The next set of questions are about your prior use and feelings towards cell phones and other types of technology. I would like to remind you that all your responses will remain confidential.

Q26a. Do you currently own a cell phone?

- ○ Yes
- ○ No

Q26b For how long have owned a cell phone?

- ○ Less than six months
- ○ 6 months to less than 1 year
- ○ 1 year to less than 2 years
- ○ 2- 3 years
- ○ More than 3 years

Q26c How much would you estimate as your average total expense on your phone in terms of airtime in a typical week?

UGX currency

____

Q26d How much would you estimate as your average total expense on your phone in terms of internet data and OTT in a typical week?

UDX currency

____

Q27a. Do you ever share your cell phone with any household member?

- ○ Yes
- ○ No

Q27b. Who do you share your phone with?

select all that apply

- [ ] Spouse
- [ ] Sibling
- [ ] Parents
- [ ] Children
- [ ] Others

please specify

____

Q28a Is your cell phone a smart phone? (i.e. A touch screen that has access to the internet and other multimedia applications)?

- ( ) Yes
- ( ) No

Q28b. What type of smartphone is it

- ( ) iPhone
- ( ) Android

Q29. For what purpose do you most frequently use your cell phone?

Select all that apply

- [ ] Making phone calls
- [ ] Sending text messages
- [ ] Sending and receiving mobile money
- [ ] Social media such Facebook, WhatsApp
- [ ] Taking picture or recording videos

Q30 How many people in your household (not counting yourself) currently own a cell phone?

- ( ) None
- ( ) 1
- ( ) 2
- ( ) 3 or more

Q31 In the last 3 months, how often have you used the internet on a smartphone?

- ( ) Daily
- ( ) 1-3 days per week
- ( ) 4-6 days per week
- ( ) Less than once per week
- ( ) Never

Q32. How easy is it for you to use the internet feature on a smartphone?

- ◯ Very Easy
- ◯ Easy
- ◯ Difficult
- ◯ Very difficult

Q33 In the last 3 months, how often have you used a social networking site (e.g., Facebook, WhatsApp, Twitter, Instagram, etc.) on a smartphone?

- ◯ Daily
- ◯ 1-3 days per week
- ◯ 4-6 days per week
- ◯ Less than once per week
- ◯ Never

Q34 In the last 3 months, how often have you used WhatsApp on a smartphone?

- ◯ Daily
- ◯ 1-3 days per week
- ◯ 4-6 days per week
- ◯ Less than once per week
- ◯ Never

Q35. How easy is it for you to use WhatsApp on a smartphone?

- ◯ Very Easy
- ◯ Easy
- ◯ Difficult
- ◯ Very difficult

Q36 In the last 3 months, how frequently have you used a cell phone to make and receive phone calls?

- ◯ Daily
- ◯ 1-3 days per week
- ◯ 4-6 days per week
- ◯ Less than once per week
- ◯ Never

Q37 How easy is it for you to use a cell phone to make and receive phone calls?

- ◯ Very easy
- ◯ Easy
- ◯ Difficult
- ◯ Very difficult

Q38 In the last 3 months, how often have you used text messaging feature on a cell phone?

- ◯ Daily
- ◯ 4-6 days per week
- ◯ 1-3 days per week
- ◯ Less than once per week
- ◯ Never

Q39 How easy is it for you to use the text messaging feature on a cell phone?

- ◯ Very easy
- ◯ Easy
- ◯ Difficult
- ◯ Very difficult

Q40. In the last 3 months, how often have you taken photos with a cell phone?

- ◯ Daily
- ◯ 4-6 days per week
- ◯ 1-3 days per week
- ◯ Less than once per week
- ◯ Never

Q41 How easy is it for you to take photos with a cell phone?

- ◯ Very easy
- ◯ Easy
- ◯ Difficult
- ◯ Very difficult

Q42. In the last 3 months, how often have you taken videos with a cell phone?

- ◯ Daily
- ◯ 4-6 days per week
- ◯ 1-3 days per week
- ◯ Less than once per week
- ◯ Never

Q43. How easy is it for you to take videos with a cell phone?

- ◯ Very easy
- ◯ Easy
- ◯ Difficult
- ◯ Very difficult

Q44. How worried would you be about losing a cell phone over the next six months if we gave you one to use?

- ◯ Not at all worried
- ◯ Somewhat worried
- ◯ Extremely worried
- ◯ Don't Know

Q45. How worried are you about someone stealing a cell phone over the next six months if we gave you one to use?

- ◯ Not at all worried
- ◯ Somewhat worried
- ◯ Extremely worried
- ◯ Don't Know

Section E: Participant transportation and other costs

Participant transportation and other costs

The next set of questions are about how your transportation costs to and from the TB clinic, and other costs you may incur due to your TB disease.

Q46. Did a family member or friend accompany you to the TB clinic for this appointment?

- ◯ Yes
- ◯ No

Q47. When coming to this TB clinic appointment, what type of transportation did you use?
select all that apply

- ☐ Personal Car
- ☐ Special hire/Uber
- ☐ Bus/taxi
- ☐ Boda Boda
- ☐ Walk
- ☐ Bicycle
- ☐ Other

please specify

____

Q48. Using this means of transportation to visit the TB clinic, estimate how long it took you to get here today?

- ◯ minutes
- ◯ Hours

please enter the minutes

____

Please enter hours

____

Q49. Approximately how much money did you spend on transport from your home to the clinic?

____

Q50. Were you accompanied by another person to the clinic?

- ○ Yes
- ○ No

Section F: Personal Social History

READ: Now I am going to ask you some questions about your social and personal life to help us understand how people do on their TB treatment. Please remember that this survey is for research purposes only. Your responses to all of these questions are confidential and will not become part of your medical record or shared with your healthcare providers.

Q51a. Do you drink alcohol?

- ○ No
- ○ Yes, but rarely like once a month
- ○ Yes, moderately at least once every week
- ○ Yes, frequently, at least every day

Q51b On average, how many alcoholic drinks do you have at a sitting? (By a drink, we mean a 350mls bottle of beer, a 150mls glass of wine, or 1 tot of liquor)

- ○ 1 drink
- ○ 2 drinks
- ○ More than 2 drinks

Q52a. Apart from the medications you take for your TB disease, do you currently take any other medications on a daily basis?

- ○ Yes
- ○ No

Q52b. For which disease do you take these medications?

select all that apply

- [ ] Hypertension
- [ ] Diabetes
- [ ] HIV/AIDs
- [ ] Malaria
- [ ] Diarrheal disease
- [ ] Respiratory tract infections
- [ ] Others

please specify

____

Q53a Do you smoke cigarettes?

- ○ No, never smoked
- ○ No, previous smoker
- ○ Yes, current smoker
- ○ None

Q53b. How many cigarettes did you/ do you smoke per day?

____

Q53c Do you smoke any other substance such as?

mark that all that apply

- [ ] Shisha
- [ ] pipe
- [ ] Marijuana
- [ ] Other

please specify

____

Q54. Generally, how often do you have difficulty swallowing pills?

- ○ Never
- ○ Rarely
- ○ Often
- ○ Always

Q55. If you were given 4 pills to swallow, how would you take them?

- One pill at a time
- A few pills at a time
- All pills at once

Q56a. What is your HIV status?

- HIV Positive
- HIV Negative
- I have never taken an HIV test
- Don't know but I have taken HIV test

Q56b. Are you currently taking antiretroviral treatment?

- Yes
- No

Q56c. How often do you take the anti-retroviral treatment?

- Once a Day
- Twice a Day
- Other

please specify

Section F: Knowledge of TB and Health Literacy

READ: Now I will ask you some questions related to what you know about TB disease and TB treatment. Your responses to all of these questions are confidential and will not become part of your medical record or shared with your healthcare providers

In brackets (Interviewer: Please note any information that a participant is missing and make sure to educate him/her)

Q57. What is the main cause of TB?

- runs in the family
- Germs
- Smoking
- Spiritual
- Others
- Don't know

please specify

Q58. How is TB spread?

Select all that apply

- [ ] Air
- [ ] Blood
- [ ] Sexual transmission
- [ ] Through vaccinations or other injections
- [ ] Direct contact with a person with TB
- [ ] Insect bite
- [ ] Food
- [ ] Don't know
- [ ] Others

please specify

Q59. How can you avoid spreading TB disease to others?

Select all that apply

- [ ] Cover mouth and nose when sneezing or coughing
- [ ] Do not share needles
- [ ] Use condoms/barriers
- [ ] Avoid touching others
- [ ] Avoid insect bites/ Sleep under mosquito nets
- [ ] Do not share food or utensils with others
- [ ] Do not share clothes with others
- [ ] Washing hands
- [ ] None
- [ ] Don't know

please specify

Q60. What are the main symptoms of pulmonary TB?

Select all that apply

- ☐ loss of appetite
- ☐ GI upset (Diarrhea/ vomiting)
- ☐ Chronic cough
- ☐ weight loss
- ☐ fever
- ☐ Night sweats
- ☐ Others
- ☐ Don't know

please specify

Q61. What are the common side effects of TB medications?

Select all that apply

- ☐ Rash
- ☐ Yellowing eyes or skin
- ☐ Body pains and joint aches
- ☐ Feeling of pins and needles in the hands and feet
- ☐ Orange discoloration of urine or other body fluids
- ☐ Don't know

Q62. What should you do if you experience side effects of your TB medications?

- ○ Stop taking my medications
- ○ Stop taking my medications and consult my doctor or health worker
- ○ Continue taking my medications
- ○ Continue taking my medications and consult my doctor or health worker
- ○ Other
- ○ Don't know

please specify

Q63. What is the total duration of pulmonary TB treatment?

- ◯ 2 months
- ◯ 6-8 months
- ◯ Life-long
- ◯ Don't know
- ◯ Other

please specify

____

Q64. When can you stop taking TB medications?

- ◯ When I feel healthy
- ◯ When my TB symptoms disappear
- ◯ When a doctor or TB worker confirms that I am cured
- ◯ Never; it is taken for life
- ◯ Don't know

Q65. What do you think will happen if you stop taking your TB medications before you complete the full course of treatment?

- ◯ I will be fully cured with no more symptoms
- ◯ I will not be cured but I will not have any more symptoms
- ◯ I will not be cured but I will no longer be able to transmit the disease to others
- ◯ The TB drugs will no longer work well on my disease
- ◯ I will die
- ◯ Don't know

Section G: Privacy, Confidentiality and Data Security Concerns

READ: Now I am going to ask you some questions about any privacy concerns you might have regarding participating in this study. Please remember that this survey is for research purposes only. Your responses to all of these questions are confidential and will not become part of your medical record or shared with your healthcare providers.

Q66a. Do you have any fears or concerns about participating in this study?

- ◯ Yes
- ◯ No

Q66b What are your concerns about participating in this study?

select all that apply

- ☐ Confidentiality and privacy of my video information
- ☐ Use of my location/ GPS data
- ☐ Difficulty in taking/sending video
- ☐ Limited interaction with health provider concerning symptoms and other problems
- ☐ Difficulty keeping the smart phone securely
- ☐ Lack of access to electricity to charge the smartphone battery
- ☐ Lack of money to pay for the internet data
- ☐ Other

please specify

____

Q67 When you have a question for your TB provider, what methods would you prefer to use to communicate with him/her?

select all that apply

- ☐ Text Message
- ☐ Whatsapp/Other messenger apps
- ☐ Phone Call
- ☐ In person
- ☐ Not Sure

Q68 If a Treatment supporter comes to supervise you taking medications at home or workplace how would you feel?

- ○ Embarrassed
- ○ Put down
- ○ Like I'm not trustworthy
- ○ Cared for/supported
- ○ I don't mind
- ○ Other

please specify

____

Q69a The Uganda National TB Program requires a Treatment supporter to observe all patients while they are taking their medication. If you are asked to be observed in-person or asked to send a video of yourself taking medications, which of the two options of observation would you prefer?

- ○ In-person DOT
- ○ Video DOT
- ○ No preference - either in person DOT or VDOT

Q70b Please tell me your reason for your choice for the previous question.

____

Section H: Family, Friend and Community Support

READ: The following questions are to understand your current family and friend community support system as a person with tuberculosis, about to start treatment. Please remember that this survey is for research purposes only. Your responses to all of these questions are confidential and will not become part of your medical record or shared with your healthcare providers

Q71. Did you have a good relationship with your household members before your TB diagnosis?

- ◯ Yes
- ◯ No

Q72. Do you feel afraid or ashamed of telling any family or household members about your TB diagnosis?

- ◯ Yes
- ◯ No

Q73. If your family or household members find out about your TB diagnosis, how do you think it will affect your relationship with them?

- ◯ Relationship will be the same, and they will show more support and care
- ◯ Relationship will Not be the same, they will not show me support and care
- ◯ They will keep their distance from me, but still show support and care
- ◯ They will keep their distance from me, and no longer show support and care

Q74a Do you think it would it be difficult for you to ask your family or household members for the support and care you need because you have TB?

- ◯ Yes
- ◯ No
- ◯ Don't know

Q74b What type of support are you likely to need from the most from family household members to complete your treatment given the list below?

Select all that apply

- ☐ Emotional (e.g. encouragement, listening ear)
- ☐ Informational (e.g. reminder to take your medications)
- ☐ Material (food, money etc)
- ☐ Transportation
- ☐ Domestic (child-care, house work etc)

Q75. Are you willing to disclose your TB diagnosis to your spouse or family household members?

- ◯ Yes
- ◯ No

Q76. Explain why you are not willing to disclose your TB status

____

READ: The following questions are to understand your current community or social support system as a person with tuberculosis, about to start treatment. This does not include your friends, family or the hospital/ research staff. Instead we are referring to other members in your community such as your work mates, schoolmates, neighbors, religious group members etc. Your responses to all of these questions are confidential and will not become part of your medical record or shared with your healthcare providers

Q77. Are you worried about other people in your community finding out about your TB diagnosis?

- ◯ Yes
- ◯ No

Q78. How would you feel if anyone in the community found out about your TB diagnosis?

- ◯ Ashamed/ embarrassed
- ◯ Worried about being rejected
- ◯ Indifferent
- ◯ People may feel less of me then make indifferent or less concerned to be option 4
- ◯ Other

please specify

____

Q79. Would you be comfortable taking your TB medicine in the presence of any person from your community?

- ◯ Yes
- ◯ No

Q80a Do you think that people in your community would offer any needed support to you even if they know you have TB disease?

- ◯ Yes
- ◯ No
- ◯ Don't know

Q80b. Which of the following types of support would you need from the community to help you take your TB treatment and complete it?

(read the options and select all that apply)

- ☐ Emotional (e.g. encouragement, listening ear)
- ☐ Informational (e.g. reminder to take your medications)
- ☐ Material (food, money etc)
- ☐ Transportation
- ☐ Domestic (child-care, house work etc)

Now we would like you to think about the community where you live. I will read some statements aloud, and ask you to judge how much you agree or disagree.

Q81. Some people may not want to eat or drink with friends who have TB

- Strongly agree
- Agree
- Disagree
- Strongly disagree

Q82. Some people feel uncomfortable about being near a person who has had TB

- Strongly agree
- Agree
- Disagree
- Strongly disagree

Q83. Some people do not want those with TB playing with their children

- Strongly agree
- Agree
- Disagree
- Strongly disagree

Q84. Some people keep their distance from people with TB

- Strongly agree
- Agree
- Disagree
- Strongly disagree

Q85. Some people do not want to talk to others with TB

- Strongly agree
- Agree
- Disagree
- Strongly disagree

Q86. Some people may not want to eat or drink with family members who have TB

- Strongly agree
- Agree
- Disagree
- Strongly disagree

Q87. Prefer not to have people with TB living in their community

- ◯ Strongly agree
- ◯ Agree
- ◯ Disagree
- ◯ Strongly disagree

Q88. Have you been tested for COVID-19 / Corona Virus?

- ◯ Yes
- ◯ No

Q89.Would you be willing to be tested for COVID-19 ?

- ◯ Yes
- ◯ No

Q90. Would you be willing to receive a vaccine for COVID-19 or Corona Virus if a vaccine is available?

- ◯ Yes
- ◯ No

You are not eligible for this study

End time and date of interview

yyyy-mm-dd ____________ hh:mm ____________

Interviewer's Initials: First and Surname

____
