# Supplementary material for: Using a Mobile Health Intervention (DOT Selfie) With Transfer of Social Bundle Incentives to Increase Treatment Adherence in Tuberculosis Patients in Uganda: Protocol for a Randomized Controlled Trial
Source: JMIR Res Protoc. 2021 Jan 5;10(1):e18029. doi: 10.2196/18029 (PMC7815451; doi:10.2196/18029)
Supplement: Multimedia Appendix 2 [file resprot_v10i1e18029_app2.docx]

**DOT Selfie Study: UC_DOT Follow-up Form (English) version 1.6**

READ: Thank you for your continued voluntary participation in the DOT Selfie study. My name is [NAME of Interviewer], and I work for the Makerere University and University of Georgia in partnership with the TB program. To remind you, we are evaluating ways of monitoring and supporting patients while they take their TB medications by using either mobile phone videos or a health worker watching a person face-to-face. In this follow-up interview we would like to ask some questions about your experiences using usual care DOT so far. Do you have any questions? [If yes, answer questions] Okay, let's begin.

Enter date of interview

Enter start time of interview

**Participant Information**

Q1 Please Enter Participant ID Number**Enter 5-Digit Number*

Q2 Please Select Site of Interview*

- Lubaga clinic
- Kitebi clinic
- Kawaala Clinic
- Other

READ: I will be asking you a set of questions today, similar to those asked at the start of the study. Each set of questions will take about 30 minutes of your time to answer. Some of the questions may seem personal, but your answers will be kept confidential. The information we gather from this interview will not be used to identify you individually, but will be combined with answers from about 140 other participants. Additionally, none of the information you provide today will be shared with your physician, nurse or TB case manager and it will not become part of your medical record. Do you have any questions? [If yes, answer questions.]

READ: The first set of questions are about how your transportation costs to and from the TB clinic, and other costs you may incur due to your TB disease. I want to remind you that your answers will be kept confidential. The information we gather from this interview will not be used to identify you individually.

Q3. Does a family member or friend accompany you to the TB clinic for your appointments?*

- Yes
- No

Q4 When coming to the TB clinic appointment, what type of transportation would you commonly use?*

- Personal Car
- Special hire/Uber
- Bus/taxi
- Boda Boda
- Walk
- Other

Q5 Using this means of transportation to visit the TB clinic, how long will it take you to get there (in minutes) from the place where you live?**Input Minutes*

Q6 Approximately how much money would you have to spend in transportation for round-trip every time you have to come to the TB clinic? If you are accompanied by someone, include their round trip costs as well.*

- UgShs
- No personal costs to me
- Don't know

Q7 How often do you have difficulty with transportation to the TB clinic?*

- Never
- Rarely
- Sometimes
- Often
- Always

Q8 Once at the TB clinic, about how long does it take you to complete your visit in minutes?**In put minutes*

Q9a Do you ever have to forfeit your work responsibilities (including absenteeism from work) because you have to meet with the TB Program Staff member to take your medications?*

- Yes
- No

Q9b How often did this happen?*

- Daily4-6 days per week
- 1-3 days per week
- Less than once per week
- Never

Q10a In the past 2 months, did you spend any money on airtime regarding your TB treatment or wellbeing (e.g. to call/text a TB healthcare provider or to upload your VDOT videos)?*

- Yes
- No

Q10b Over the past 2 months, on average how many times did you spend money on airtime minutes regarding your TB treatment or wellbeing as described above?*

- Daily4-6 days per week
- 1-3 days per week
- Less than once per week
- Never

Q10c On average over the past two months, what was your total airtime cost regarding your TB treatment or wellbeing as described above?**UGShs*

READ: The next set of questions are related to your TB treatment experiences while you were on in-person DOT. Again, let me remind you that your answers are confidential and that they will not be shared with your physician or the TB Control program. Your honest answers will help us improve the in-person DOT System.

Q11 Overall, how satisfied are you with the service you have received so far for your TB treatment?*

- Very Dissatisfied
- Dissatisfied
- Indifferent
- Satisfied
- Very Satisfied

Q12 How satisfied are you with the clinical care you received by the health provider at the TB clinic?*

- Very Dissatisfied
- Dissatisfied
- Indifferent
- Satisfied
- Very Satisfied

Q13 How satisfied were you with your TB treatment monitoring using in-person DOT? (Choose one)*

- Very Dissatisfied
- Dissatisfied
- Indifferent
- Satisfied
- Very Satisfied

Q14 How would you compare in-person DOT to VDOT?*

- In-person was better than VDOT
- No difference
- VDOT was better than in-person DOT

Q15 How do you feel about the amount of contact you have with the TB program staff during in-person DOT?*

- Too much contact
- Just enough contact
- Not enough contact

Q16 What is your preferred location to take your TB medications*

- Home
- Work
- School
- Other
- No preference

Q17 How often were you away from your preferred location when you met with the TB Program staff member to take your TB medications?*

- Daily4-6 days per week
- 1-3 days per week
- Less than once per week
- Never

Q18 How important was it for you to be able to take your medications at your preferred location using in-person DOT treatment?*

- Not at all important
- Somewhat important
- Very Important

Q19 How important was it for you to be able to choose the time of day that you took your medications using in-person DOT treatment?*

- Not at all important
- Somewhat important
- Very Important

Q20 How often were you able to choose the time of day to meet with the TB Program staff member for your in-person DOT treatment? [Read responses] (Choose One)*

- Daily
- 4-6 days per week
- 1-3 days per week
- Less than once per week
- Never

Q21 Based on your experience with in-person DOT so far, if you had to redo the TB treatment, would you choose in-person DOT or VDOT?*

- VDOT
- No preference
- In-person DOT

Q22 Please tell me your reason for your choice for the previous question.*Q23 Would you recommend in-person DOT to other TB patients?*

- Yes
- No

READ: Now I am going to ask you some questions about your experiences when using the the in-person DOT method to take your medications. Your answers are confidential and that they will not be shared with your physician or the TB Control program.Q24 Overall, how difficult did you find the in-person DOT process? (For example, finding a good time to meet with the TB Program staff member, finding a private place to meet the TB Program staff member etc.)*

- Very Difficult
- Difficult
- Easy
- Very Easy

Q25 Did you get TB drugs at every visit from the TB Program staff member?*

- Yes
- No

Q26a How many days each week were you able to meet with the TB Program staff member for your in-person DOT? [Read responses]*

- Daily4-6 days per week
- 1-3 days per week
- Less than once per week
- Never

Q26b What were the main reasons for not meeting with the TB Program staff member? [Read responses]**Select all that apply*

- TB Program staff member not friendly
- Could not find an appropriate time or placeI had side effects of TB drugs
- I felt cured / healedI did not know we had to meet daily
- We could not meet at the weekends
- Other reasons

Q26c Did you still take your medications on days you were unable to meet with TB Program staff member?*

- Yes
- No

Q26d Did you have a friend/family observe you take your TB medications when you were unable to meet with the TB Program staff member?*

- Yes
- No

Q27 Can you tell me all places you met with the TB Program staff member to take your mediations, even if it was only once?**Select all that apply*

- Home
- Work
- Car
- School
- Other

Q28 In the past 2 months, where were you most often? (Choose one)*

- Home
- Work
- School
- Other

Q29 How often did you get encouragement/ motivation from the TB Program staff person to follow the drug schedule? [Read responses] (Choose One)*

- Daily
- 4-6 days per week
- 1-3 days per week
- Less than once per week
- Never

Q30 Did you find this encouragement/ motivation useful to follow the drug schedule?*

- Yes
- No

Q31 How often did you get health education from the TB Program staff person? [Read responses] (Choose One)*

- Daily
- 4-6 days per week
- 1-3 days per week
- Less than once per week
- Never

Q32 How often did the TB Program staff person ask about any side effects you were experiencing from TB drugs? [Read responses] (Choose One)*

- Daily
- 4-6 days per week
- 1-3 days per week
- Less than once per week
- Never

Q33a Did you experience any side effects while on your TB medications?*

- Yes
- No

Q33b What side effects did you experience?**Select all that apply*

- Rash
- Yellowing eyes or skin
- Body pains and joint aches
- Numbness
- Orange discoloration of urine or other body fluids
- Other

Q33c In what ways did you manage or relieve your side effects?*

- I stopped taking my medications to relieve my side effects
- I took my medications with food to relieve the side effects
- I took my medications at specific times of the day to relieve the side effects
- I took another medication with my TB medications to relieve the side effects
- Other

Q34a Apart from the medications you take for your TB disease, do you currently take any other medications on a daily basis?*

- Yes
- No

Q34b For which disease do you take these medications?**Select all that apply*

- HypertensionDiabetes
- HIV/AIDsMalaria
- Diarrheal disease
- Respiratory tract infections
- Others

Q35 Generally, how often do you have difficulty swallowing pills?*

- Never
- Rarely
- Often
- Always

Q36 Generally, how do you take your pills?*

- One pill at a time
- A few pills at a time
- All pills at once

Q37 How often did you need to consult a health provider in relation to TB treatment while using in-person DOT? (Choose one)*

- Daily
- 4-6 days per week
- 1-3 days per week
- Less than once per week
- Never

READ: The following questions are about the TB and DOT training you had at the beginning of the study. Please be assured that your answers will be kept confidential and your health care provider will not see your responses

Q38 Did you find the in-person DOT training process helpful?*

- Yes
- No

Q39 Was the explanation of possible symptoms and side effects of the TB medications made clear to you?*

- Yes
- No

Q40 What changes would you suggest to improve the patient training process for standard DOT?*

READ: The following questions are about the any privacy concerns you might have regarding the VDOT study. Please be assured that your answers will be kept confidential and your health care provider will not see your responses

Q41 How important was it to you to meet the TB Program staff member in private?*

- Very Important
- Somewhat Important
- Not Important

Q42 Were you concerned with privacy whilst taking your medications using in-person DOT any point during the study?*

- Yes
- No

Q43 Did you have any difficulty finding a private place to meet the TB Program staff member?*

- Yes
- No

Q44 Did you find the in-person DOT process more, less or just as confidential as VDOT?*

- More
- Less
- Same

Q45 Were you ever concerned about people seeing you take your medication using in-person DOT?*

- Yes
- No

Q46 Were you ever questioned by anyone about taking your TB medications while using the in-person DOT method*

- Yes
- No

Q47 Did you ever share your experiences about participating in the VDOT study with your family?*

- Yes
- No

Q48 Did you ever share your experiences about participating in the VDOT study with your friends, neighbors, schoolmates or workmates?*

- Yes
- No

Q49 Did you ever fail to meet with the TB Program staff member because you were afraid someone might see you taking your medicine?*

- Yes
- No

Q50 Lastly, we are interested in capturing personal stories about our participants' experiences using in-person DOT. Is there anything you would like to share with us about how in-person DOT made your treatment easier or harder? What do you think other patients with TB, doctors or the public should know-good or bad-about using in-person DOT for helping patients to take all of their medications? Or any general comment about your experience on in-person DOT?*

- Yes
- No

READ: This concludes our interview today. Thank you for participating in the VDOT study and for completing this follow-up interview. Please be assured that your answers will be kept confidential and your health care provider will not see your responses. You will receive a transport reimbursement from the research nurse for completing today's visit. We appreciate your feedback as this information will help us improve the TB program for future patients. Do you have any questions?*

- Yes
- No

End date of interview

End time a of interview

Interviewer's Initials: First and Surname
