# Supplementary material for: Using a Mobile Health Intervention (DOT Selfie) With Transfer of Social Bundle Incentives to Increase Treatment Adherence in Tuberculosis Patients in Uganda: Protocol for a Randomized Controlled Trial
Source: JMIR Res Protoc. 2021 Jan 5;10(1):e18029. doi: 10.2196/18029 (PMC7815451; doi:10.2196/18029)
Supplement: Multimedia Appendix 3 [file resprot_v10i1e18029_app3.docx]

**DOT Selfie_VDOT Follow-Up Form English**

**version 1.6**

READ: Thank you for your continued voluntary participation in the DOT Selfie study. My name is [NAME of Interviewer], and I work for the Makerere University and University of Georgia in partnership with the TB program. To remind you, we are evaluating ways of monitoring and supporting patients while they take their TB medications by using either mobile phone videos or a health worker watching a person face-to-face. In this follow-up interview we would like to ask some questions about your experiences using VDOT so far. Do you have any questions? [If yes, answer questions] Okay, let's begin.

Enter date of interview*

Enter time of interview*

Q1. Please Enter 5-Digit Participant Study ID*

Q2 Please Enter 10-Digit Study Assigned Phone Number

Q3 Please Select Site of Interview*

- Lubaga clinic
- Kitebi clinic
- Kawaala
- ClinicOther

READ: I will be asking you a set of questions today, similar to those asked at the start of the study. Each set of questions will take about 30 minutes of your time to answer. Some of the questions may seem personal, but your answers will be kept confidential. The information we gather from this interview will not be used to identify you individually, but will be combined with answers from about 140 other participants. Additionally, none of the information you provide today will be shared with your physician, nurse or TB case manager and it will not become part of your medical record. Do you have any questions? [If yes, answer questions.]Participant InformationREAD: The first set of questions are about your prior use and feelings towards cell phones and other types of technology. I would like to remind you that all your responses will remain confidential.

Q4 In the last 2 months, how often have you used the internet on a smartphone?*

- Daily4-6 days per week
- 1-3 days per week
- Less than once per week
- Never

Q5 How easy is it for you to use the internet feature on a smartphone?*

- Very Easy
- Easy
- Difficult
- Very difficult

Q6 In the last 2 months, how often have you used any social networking site (e.g., Facebook, WhatsApp, Twitter, Instagram, etc.) on a smartphone?*

- Daily4-6 days per week
- 1-3 days per week
- Less than once per week
- Never

Q7 In the last 2 months, how often have you used WhatsApp on a smartphone?*

- Daily4-6 days per week
- 1-3 days per week
- Less than once per week
- Never

Q8 How easy is it for you to use WhatsApp on a smartphone?*

- Very Easy
- Easy
- DifficultVery
- difficult

Q9 In the last 2 months, how often have you used a cell phone to make and receive phone calls?*

- Daily4-6 days per week
- 1-3 days per week
- Less than once per week
- Never

Q10 How easy is it for you to use a cell phone to make and receive phone calls?*

- Very easy
- Easy
- Difficult
- Very difficult

Q11 In the last 2 months, how often have you used text messaging feature on a cell phone?*

- Daily
- 4-6 days per week
- 1-3 days per week
- Less than once per week
- Never

Q12 How easy is it for you to use the text messaging feature on a cell phone?*

- Very easy
- Easy
- DifficultVery
- difficult

Q13 In the last 2 months, how often have you taken photos with a cell phone?*

Daily

- 4-6 days per week
- 1-3 days per week
- Less than once per week
- Never

Q14 How easy is it for you to take photos with a cell phone?*

- Very easy
- Easy
- Difficult
- Very difficult

Q15 In the last 2 months, how often have you taken videos with a cell phone?*

- Daily4-6 days per week
- 1-3 days per week
- Less than once per week
- Never

Q16 How easy is it for you to take videos with a cell phone?*

- Very easy
- Easy
- DifficultVery
- difficult

READ: The next set of questions are about how your transportation costs to and from the TB clinic, and other costs you may incur due to your TB disease. I want to remind you that your answers will be kept confidential. The information we gather from this interview will not be used to identify you individually.

Q17. Has a family member or friend accompanied you today to the TB clinic for your appointments?*

- Yes
- No

Q18 When coming to the TB clinic appointment, what type of transportation would you commonly use?*

- Personal Car
- Special hire/Uber
- Bus/taxi
- Boda Boda
- Walk
- Other

Q19 Using this means of transportation to visit the TB clinic, estimate the time (in minutes) you spent to travel to this TB clinic from where you live?*

Q20 Approximately how much money do you have to spend in transportation for round-trip each time you have to come to the TB clinic? If you are accompanied by someone, include their round trip costs as well.*

- UgShs
- No personal costs to me
- Don't know

Q21 How often do you have difficulty with transportation to the TB clinic?*

- Never
- Rarely
- Sometimes
- Often
- Always

Q22 Once at the TB clinic, estimate how much time (in minutes) it takes you to complete your visit?*……………….

Q23a Do you ever have to forfeit your work responsibilities (including absenteeism from work) because you have to meet with your DOT treatment supporter to take your medications?*

- Yes
- No

Q23b How often did this happen?*

- Daily
- 1-3 days per week
- 4-6 days per week
- Less than once per week
- Never

Q24a In the past 2 months, did you spend any money on airtime regarding your TB treatment or wellbeing (e.g. to call/text a TB healthcare provider or to upload your VDOT videos)?*

- Yes
- No

Q24b Over the past 2 months, on average how many times did you spend money on airtime minutes regarding your TB treatment or wellbeing as described above?*

- Daily
- 4-6 days per week
- 1-3 days per week
- Less than once per week

Q24c On average over the past two months, estimate how much your total airtime cost was regarding your TB treatment or wellbeing as described above?**input UGX*

READ: The next set of questions are related to your TB treatment experiences while you were on VDOT. Again, let me remind you that your answers are confidential and that they will not be shared with your physician or the TB Control program. Your honest answers will help us improve the VDOT System.

Q25 Overall, how satisfied are you with the service you have received so far for your TB treatment?**choose one*

- Very Dissatisfied
- Dissatisfied
- Indifferent
- Satisfied
- Very Satisfied

Q26 How satisfied are you with the clinical care you received by the health provider at the TB clinic?**choose one*

- Very Dissatisfied
- Dissatisfied
- Indifferent
- Satisfied
- Very Satisfied

Q27 How satisfied were you with your TB treatment monitoring using VDOT? (Choose one)**choose one*

- Very Dissatisfied
- Dissatisfied
- Indifferent
- Satisfied
- Very Satisfied

Q28 How would you compare in-person DOT to VDOT? [Read responses] (Choose one)*

- VDOT was better than in-person DOT
- No difference
- In-person was better than VDOT

Q29 How do you feel about the amount of contact you had with the TB program staff during VDOT?**Choose One*

- Too much contact
- Just enough contact
- Not enough contact

Q30 What is your preferred location to record yourself taking your TB medications [Read responses]*

- Home
- Work
- School
- Other
- No preference

Q31 How often were you away from your preferred location when you recorded your videos? [Read responses] (Choose one)*

- Daily
- 4-6 days per week
- 1-3 days per week
- Less than once per week
- Never

Q32 How important was it for you to be able to record videos (whilst taking your TB medications) at your preferred location?*

- Not at all important
- Somewhat important
- Very Important

Q33 How important was it for you to be able to choose the time of day that you took your medications using VDOT?*

- Not at all important
- Somewhat important
- Very Important

Q34 Based on your experience with VDOT so far, if you had to redo the TB treatment, would you choose in-person DOT or VDOT? (Choose one)*

- VDOT
- No preference
- In-person DOT

Q35 Please tell me your reason for your choice for the previous question.*Q36 Would you recommend VDOT to other TB patients?*

- Yes
- No

READ: Now I am going to ask you some questions about your experiences when using the phone to record your videos. Your answers are confidential and that they will not be shared with your physician or the TB Control program.Q37 Overall, how difficult did you find the VDOT process? (For example, recording yourself and sending videos) (Read options) (Choose one)

- Very Difficult
- Difficult
- EasyVery
- Easy

Q38 Since you started VDOT, how often did you use the VDOT phone for personal use (for example to make or receive phone calls or to use the internet)? It is okay if you did, we just want to know what other phone functions were used.

- Daily
- 1-3 days per week
- 4-6 days per week
- Less than once per week
- Never

Q39a When using the phone for personal use, what functions on the phone did you use?*

- Text messaging
- Internet
- Email
- Phone Calls
- Games
- Watching Videos
- Taking photos
- Alarm/Calendar
- Other

Q39b How often did you take the VDOT phone with you when you left your home? (Choose one)*

Daily

- 4-6 days per week
- 1-3 days per week
- Less than once per week
- Never

Q40 Can you tell me all places you recorded your videos, even if it was only once?**Check all that apply*

- Home
- Work
- Car
- School
- Other

Q41 In the past 2 months, where were you most often? (Choose one)*

- Home
- Work
- School
- Other

Q42 Did you ever fail to record a dose of medication because your phone was not charged?*

- Yes
- No

Q43 How often did you have problems with the VDOT application (Choose One)*

- Daily
- 4-6 days per week
- 1-3 days per week
- Less than once per week
- Never

Q44 How often did you ever have a family member, friend, or other person help you record your video (Choose One)*

- Daily
- 4-6 days per week
- 1-3 days per week
- Less than once per week
- Never

Q45 Did you ever receive text messages that reminded you to take your medication?*

- Yes
- No

Q48 How many text reminders (per week) do you think would be enough?**Please Enter Number of Texts*

Q49 Did you ever text your provider with the study cell phone?*

- Yes
- No

Q50 How often did you get health education from a staff member of the TB Program? [Read responses] (Choose One)*

- Daily
- 4-6 days per week
- 1-3 days per week
- Less than once per week
- Never

Q51 How often did a staff member of the TB Program ask about any side effects you were experiencing from TB drugs? [Read responses] (Choose One)*

- Daily
- 4-6 days per week
- 1-3 days per week
- Less than once per week
- Never

Q52 How often did you have problems uploading a video due to poor network reception? (Choose One)*

- Daily
- 4-6 days per week
- 1-3 days per week
- Less than once per week
- Never

Q53 How often did you have to change/move locations due to poor network reception?*

- Daily
- 4-6 days per week
- 1-3 days per week
- Less than once per week
- Never

Q54a Did you ever record a video while you were traveling outside of your residence?*

- Yes
- No

Q55b How often were you able to record your videos while you were traveling? (Choose one)*

- Never
- Rarely
- Sometimes
- Mostly
- Always

Q56 How often did you need to consult a health provider in relation to TB treatment while using VDOT? (Choose one)*

- Daily
- 4-6 days per week
- 1-3 days per week
- Less than once per week
- Never

Q57a Did you experience any side effects while on your TB medications?*

- Yes
- No

Q59a Apart from the medications you take for your TB disease, do you currently take any other medications on a daily basis?*

- Yes
- No

Q60Generally, how often do you have difficulty swallowing pills?*

- Never
- Rarely
- OftenA
- lways

Q61a Generally, how do you take your pills?*

- One pill at a time
- A few pills at a time
- All pills at once

Q61b Generally, what do you use to take your pills?*

- Water/tea/ porridge/juice
- Insert pills in banana or any other fruit
- Crush the tablets
- Other

Q62a In future studies, if you had a choice of using your own cell phone or the one loaned to you for VDOT, which would you prefer? (Choose one)*

- Study Phone
- Personal Phone
- No Preference

Q62b Please tell me your reason for your choice for the previous question

READ: The following questions are about the TB and VDOT training you had at the beginning of the study. Please be assured that your answers will be kept confidential and your health care provider will not see your responsesQ63 Did you find the VDOT training process helpful?*

- Yes
- No

Q64 Did you ever use any of the contact numbers listed on the information packet?*

- Yes
- No

Q65 Was the explanation of possible symptoms and side effects of the TB medications made clear to you?*

- Yes
- No

Q66 What changes would you suggest to improve the patient training process for VDOT?*

READ: The following questions are about any privacy concerns you might have regarding the VDOT study. Please be assured that your answers will be kept confidential and your health care provider will not see your responses.

Q67 How important was it to you to be able to record your videos in private? (Choose one)*

- Extremely important, could not record without privacy
- Somewhat, Important, but privacy doesn't prevent video recording
- Not Important, don't mind whether recording in a public or private place

Q68 Were you concerned with the privacy of your videos at any point during the study?*

- Yes
- No

Q69 Did you have any difficulty finding a private place to record your video?*

- Yes
- No

Q70 Did you find the VDOT process more, less or the same level of confidential than in-person DOT? (Choose one)*

- More
- Less
- Same

Q71 Were you ever concerned about people seeing you make your VDOT video?*

- Yes
- No

Q72 Were you ever questioned by anyone about you making VDOT videos?*

- Yes
- No

Q73 Did you ever share your experiences about participating in the VDOT study with your family?*

- Yes
- No

Q74 Did you ever share your experiences about participating in the VDOT study with your friends, neighbors, schoolmates or workmates?*

- Yes
- No

Q75 Did you ever fail to record a video because you were worried someone else might see you taking your medicine and recording a video?*

- Yes
- No

Q76 Do you still have the loaned smartphone in your possession? (i.e. it is not missing/ has not been stolen)*

- Yes
- No

Q77 Lastly, we are interested in capturing personal stories about our participants' experiences using VDOT. At a later time, would you be willing to share with us about how VDOT made your treatment easier or harder?*

- Yes
- No

READ: This concludes our interview today. Thank you for participating in the VDOT study and for completing this follow-up interview. Please be assured that your answers will be kept confidential and your health care provider will not see your responses. You will receive a transport reimbursement from the research nurse for completing today's visit. We appreciate your feedback as this information will help us improve the TB program for future patients.

Q78.Do you have any questions?*

- Yes
- No

End date of interview
