# Supplementary material for: Using a Mobile Health Intervention (DOT Selfie) With Transfer of Social Bundle Incentives to Increase Treatment Adherence in Tuberculosis Patients in Uganda: Protocol for a Randomized Controlled Trial
Source: JMIR Res Protoc. 2021 Jan 5;10(1):e18029. doi: 10.2196/18029 (PMC7815451; doi:10.2196/18029)
Supplement: Multimedia Appendix 4 [file resprot_v10i1e18029_app4.docx]

1. Do you always take your TB medication at a specific time?

Yes

No

1. Would you continue taking your TB medications when you feel well?

Yes

No

1. Would you continue taking your TB medications when you feel unwell?

Yes

No

1. Do you ever forget to take your medications?

Yes

No

1. Do you always take your TB medications during the weekend?

Yes

No

1. When you travel away from home, do you always take your TB medications (and study phone, if applicable) with you?

Yes

No

1. Have you ever missed getting a TB medication refill?

Yes

No

1. Did you take your medications yesterday?

Yes

No

1. Did you miss any medication dose in the last seven days?

Yes

No

1. How many days have you ever missed your treatment dose because you had run out of TB medications? (Put zero if no doses have been missed)
