# Supplementary material for: Using a Mobile Health Intervention (DOT Selfie) With Transfer of Social Bundle Incentives to Increase Treatment Adherence in Tuberculosis Patients in Uganda: Protocol for a Randomized Controlled Trial
Source: JMIR Res Protoc. 2021 Jan 5;10(1):e18029. doi: 10.2196/18029 (PMC7815451; doi:10.2196/18029)
Supplement: Multimedia Appendix 5 [file resprot_v10i1e18029_app5.pdf]

SUMMARY STATEMENT
(Privileged Communication)

PROGRAM CONTACT:
Laura Povlich
301-496-1653
povlichlk@mail.nih.gov

Release Date: 03/20/2019
Revised Date:

Application Number: 1 R21 TW011365-01

Principal Investigator

SEKANDI, JULIET NABBUYE

Applicant Organization: UNIVERSITY OF GEORGIA

Review Group: ZRG1 HDM-A (55)
Center for Scientific Review Special Emphasis Panel
PAR-16-292 Mobile Health: Technology and Outcomes in Low and Middle Income Countries

Meeting Date: 03/01/2019
Council: MAY 2019
Requested Start: 07/01/2019
RFA/PA: PAR18-242
PCC: MHEALTH

Project Title: DOT Selfie: A Mobile Health Intervention with Transfer of Social Bundle Incentives to Increase Treatment Adherence in Tuberculosis Patients in Uganda
SRG Action: Impact Score:20 Percentile:4 #
Next Steps: Visit https://grants.nih.gov/grants/next_steps.htm
Human Subjects: 30-Human subjects involved - Certified, no SRG concerns
Animal Subjects: 10-No live vertebrate animals involved for competing appl.
Gender: 1A-Both genders, scientifically acceptable
Minority: 5A-Only foreign subjects, scientifically acceptable
Children: 3A-No children included, scientifically acceptable

1R21TW011365-01 Sekandi, Juliet

RESUME AND SUMMARY OF DISCUSSION: This application proposes to adapt and test the preliminary effectiveness of a mHealth intervention that uses video directly observed therapy (VDOT) to monitor and ensure medication adherence among patients receiving tuberculosis (TB) treatment in Uganda. During discussion the Editors noted that the proposed study addresses a significant and growing global health issue and if successful, findings will likely inform strategies to improve TB treatment outcomes in low-and middle-income countries (LMICs). The investigators are a strong team and a good match for the project. Additionally, the Committee agreed that the proposed mHealth approach is innovative and scalable. Plans to build capacity for mHealth research in Uganda are strong. There is some minor concern regarding the sustainability of the proposed incentives and it is uncertain that patients will be trained in use of self-videos. Overall, the proposed research is potentially very important and findings are expected to have a high impact on efforts to build the capacity for mHealth research in Uganda and to improve tuberculosis treatment adherence in resource-limited settings.

DESCRIPTION (provided by applicant): Tuberculosis (TB) is one of the deadliest infectious diseases and a killer of 1.7 million people worldwide in 2016, majority of whom were in low- and middle-income countries. Medication non-adherence is a common, complex, and costly problem. Non-adherence to treatment is a major obstacle to TB control: it reduces cure rates, prolongs infectiousness, and contributes to the emergence of multi-drug resistant strains of TB. Careful monitoring and support of treatment adherence is needed to mitigate the rapid emergence of MDR-TB and other adverse disease outcomes. The lack of a sufficient number of health workers and the cost of travel makes it particularly challenging to implement the current standard in-person directly observed therapy (DOT). We propose to adapt and test an existing mobile health intervention -video directly observed therapy (VDOT), for use in the local Ugandan context. The adapted intervention dubbed “DOT Selfie” is expected to provide a new alternative way for health providers to monitor patients remotely. First, “DOT Selfie” will entail a patient using a smartphone to record and send a time-stamped encrypted video showing his/her medications intake each day. This video component will directly substitute the need for daily face-to-face meetings between the provider and patient thereby minimize costly travel. Second, the patient will also receive daily SMS text reminders translated in Luganda and a weekly reward if they send their videos. This component will enhance patient- provider interaction while motivating desired adherence behavior. The provider logs on a secured computer system to verify treatment adherence. We hypothesize that, by eliminating the need for daily travel, allowing patients to take their pills anywhere and providing incentives to TB patients, DOT Selfie will increase treatment adherence. The long-term goal of this project is to establish DOT Selfie as an effective way to improve medication adherence in TB treatment in an African context. To achieve this goal, it will be critical to perform reliable research to assess effectiveness of contextualized mHealth tools and also to build a knowledgeable and competent workforce that will properly implement mHealth interventions in Africa. In partnership with Makerere University in Uganda, the Uganda National TB Program and researchers at the University of Georgia, the team will work to address the scientific and capacity building objectives through these Specific Aims. First, we will conduct qualitative interviews to identify perceived barriers to use of mHealth tools among patients and providers. Second, we will test the adapted DOT Selfie intervention for monitoring adherence among 30 TB patients compared to those in standard of care. Finally, we will use a multi-prong approach to build and strengthen the capacity for mHealth research at Makerere University and within the National TB Program in Uganda. The results will be applicable to other African settings and to a range of chronic diseases with lifelong treatment such HIV/AIDs.

PUBLIC HEALTH RELEVANCE: This project will pilot DOT Selfie, an adapted mobile-based intervention that uses video to observe pill intake by patients to improve adherence monitoring of TB treatment in Uganda. The intervention is based on Video Directly Observed Therapy (VDOT), which consists of a smartphone App for recording and sending videos to a secure server. The sent videos are

immediately accessed and viewed by health workers eliminating the need to travel to the patients' home or work place. The proposed project will innovatively add a low-cost weekly incentive in form of prepaid access to social media or voice calls to chat with their friends and family. We expect the intervention to improve adherence to TB treatment and provide a feasible alternative for the Uganda National TB Control Program that helps to overcome practical challenges of health worker shortages and costly travel.

CRITIQUE

EDITORS' IMPACT STATEMENTS:

Overall Impact 1: Directly Observable Therapy is the gold-standard for ensuring adherence to TB therapy but is expensive and very difficult to scale. This is a well-written and well-researched proposal that presents an innovative system, called Dot Selfie, in which patients are sent reminders to take their medicines and an app on their smartphone records them doing so and sends the video to a central server. Continued regular adherence to this is rewarded by credit for a "social bundle" paying for the patient to avail of various networking services on their phone. By combining reminders with rewards, the Dot Selfie system has real potential to make a scalable breakthrough advance in a very difficult problem in LMICs. One concern in the proposal is the eventual sustainability of the project beyond the study period. For example, one question is who will pay for the social bundle (or airtime), an essential part of the project, in order to put it into widespread TB patient use. While the cost in US currency is only 33 cents per week, this could be a substantial amount in Uganda especially because TB treatment can continue for months and years. Another aspect that will need attention is to train patients to ensure quality control of self-videos. Finally, it is noted that smartphones and high-speed data networks (3G to 4G) are essential to dot-selfie. However, smartphone usage is increasing greatly even in LMICs and high-speed data network infrastructure is also being added. mHealth research capacity building has been addressed very well and is likely to yield considerable benefits. Human subjects and inclusion are adequate.

Overall Impact 2: This application proposes a mobile phone-based video selfie app (VDOT) to monitor pill intake to improve drug adherence among patients receiving TB treatment in Uganda. This is a highly innovative application, which can potentially make a substantial impact on TB treatment adherence and ultimately improve TB treatment outcomes in resource-limited settings. The scientific premise includes that treatment adherence is key to the success of TB treatment; the proposed VDOT app offers a low-cost, effective adherence monitoring tool to improve TB adherence; and the overall public health impact of this application is deemed to be on that, if the proposed VDOT is proven to be effective, it will provide a valuable mHealth tool in treatment adherence. This is well written proposal with relatively high impact and no weakness.

Overall Impact 3: This project proposes to develop and test in a pilot RCT the preliminary effectiveness of a mobile health intervention, compared to the DOT selfie, in improving medication adherence in patients newly diagnosed with TB in Kampala, Uganda. The project is of great significance, given given TB remains of the deadliest infectious diseases in the world, with non-adherence being one of the main reasons that patients fail therapy and develop MDR. The advantage of the DOT selfie over DOT is that it can be easily assessible in areas where DOT is not practical, and the video DOT offers a way to overcome these barriers through the use of smartphones, which are widespread in Uganda. Despite the junior status of the PI, she was a FIC international training scholarship awardee, and has assembled an impressive investigative team with the complementary experiences/skills to successfully accomplish all study aims. The study is highly innovative in using relatively cheap incentives of social media airtime minutes to enhance medication adherence. The

approach is appropriate, with a) a qualitative study to identify potential barriers and adapt the VDOT platform to add the social bundle incentives transfer, b) a pilot RCT to test the preliminary effectiveness of the DOT selfie with incentives on TB medication adherence, and c) a program for building sustainable capacity for m-health research in Uganda. The environments at the partnering institutions are both excellent and have the resources necessary for project completion. In summary, there is every reason to believe that this project can have major impact on both TB medication adherence in Uganda, and other LMICs, as well as developing research capacity in mHealth in Uganda. Timeline: Reasonable. Human Subject concerns: None, in fact strong protections in place even though risks are low.

MAIL REVIEWERS' CRITIQUES:

CRITIQUE 4

Significance: 1
Investigator(s): 1
Innovation: 1
Approach: 1
Environment: 1

Overall Impact: The applicants propose to adapt a cellphone-based incentive system to a cellphone-based means of ensuring adherence to treatment for TB. The research builds on preliminary research showing the need for motivation to consistently use video-assisted directly observed therapy (VDOT). The proposed motivation is a reward of access to social media. The incentive is available, affordable, and conceivably effective. The applicants have a strong record of collaboration and access to potential study participants. The intervention will be assessed with a randomized assignment to the intervention and standard DOT. The applicants will also enhance the ability of Ugandan university students and faculty to engage in mHealth research, thereby increasing the utility and sustainability of their own research. This mHealth research capacity is valuable for Uganda, which is famous as a country where many mHealth applications are tested.

1. Significance:

Strengths

- Addresses an important public health disease (TB).
- Addresses an important challenge in TB control (extending the range of effective VDOT).

Weaknesses

- None.

2. Investigator(s):

Strengths

- A history of collaboration.
- Appropriately engage local researchers.

Weaknesses

- None.

3. Innovation:

Strengths

- The implementation of an imminently accessible incentive that has yet to be evaluated (a “social internet bundle”).

Weaknesses

- None.

4. Approach:

Strengths

- The evaluation is appropriate for the stage of knowledge (pilot study).

Weaknesses

- The applicants say they are not seeking statistical significance (as would be appropriate for a pilot), but then proceed to document their statistical power.

5. Environment:

Strengths

- Long history of work and collaboration with their Ugandan colleagues and institutions.

Weaknesses

- None.

Study Timeline:

Strengths

- The sample size described in the application is feasible for the study duration.

Weaknesses

- None noted.

Protections for Human Subjects:

Acceptable Risks and/or Adequate Protections

Data and Safety Monitoring Plan (Applicable for Clinical Trials Only):

Acceptable

Inclusion of Women, Minorities and Children:

- Sex/Gender: Distribution justified scientifically
- Race/Ethnicity: Distribution justified scientifically
- For NIH-Defined Phase III trials, Plans for valid design and analysis:
- Inclusion/Exclusion of Children under 18: Excluding ages <18; justified scientifically

Budget and Period of Support:

Recommend as Requested

CRITIQUE 5

Significance: 1
Investigator(s): 2
Innovation: 2
Approach: 2
Environment: 1

Overall Impact: “DOT Selfie: A mobile health intervention with transfer of social bundle incentives to increase treatment adherence in tuberculosis patients in Uganda” is a new R21 application from Juliet Sekandi from University of Georgia (US) and colleagues from University of Georgia and Makerere University in Kampala, Uganda. The proposal describes how use of a m-Health approach could help improve adherence in patients newly diagnosed with TB in Uganda. The approach is very solid beginning with a qualitative analysis of the intervention, then a small pilot RCT comparing DOT Selfie to DOT. An entire aim is devoted to training 10 key healthcare personnel. The assembled team is strong and likely to be able to perform the study as described. In Summary, this proposal which involves a small pilot RCT, is an innovative approach to potentially improving adherence to TB treatment. The many positive aspects include the qualitative upfront analysis, the small RCT, the training component and the team. There a few minor concerns such as the small sample size and somewhat limited experience of the PI. Overall, it is likely this proposal can be completed and provide preliminary results which could lead to an intervention that is practical and of value.

1. Significance:

Strengths

- TB remains one of the deadliest infectious diseases in the world. Non-adherence is the main reason patients fail therapy and develop MDR. DOT is the standard, but it is impractical in many regions.
- The use of smartphones is very high in some LMICs and video DOT or VDOT, offers a unique way of balancing the benefits of DOT remotely by use of a smartphone.

Weaknesses

- None.

2. Investigator(s):

Strengths

- Juliet Sekandi, MD, DrPH is a medical doctor and assistant professor, College of Public Health at UGA. Her fields of study include epidemiology, mobile health research and global health with a focus on TB. She will be project PI. She was a FIC international training scholarship awardee. She has published in PLoS One, Int J of TB lung dis, and BMC Public Health among others. She has been funded previously by DDCF, USAID and FHDRC.
- Chris Whalen, MD, MS is Ernest Corn Professor of ID Epi at UGA. He is a physician Epidemiologist with a focus on TB and HIV. He has received awards for his work in Uganda. Well published and funded by FIC and NIAID.
- Kevin Dobbin, PhD is Associate Professor of Biostatistics at UGA. He will be co-I. He has many relevant publications and past funding from NIH.
- Esther Buregyeya, MD, PhD is Senior Lecturer and Chair of the Department of Disease Control & Environmental Health at Makerere. She will implement the study in Uganda and will be co-PI.
- Lynn Aluyambe, PhD MPH is Associate Professor of Community Health and Behavioral Sciences at Makerere. She is a social-behavioral scientist and have been co-I on an m-Health

project to improve communication between village health team and mothers with newborns in rural Uganda. She has many relevant publications and is funded by FIC (U01).

Weaknesses

- The PI and Dr Dobbin have no active external funding at present.

3. Innovation:

Strengths

- DOT Selfie is an VDOT that goes beyond what's been done elsewhere, specifically, the incentive of airtime minutes is quite novel and likely to be accepted; and customized SMS text reminders.

Weaknesses

- Text adherence reminders already have a long history with mixed results.

4. Approach:

Strengths

- The goal of this proposal is to use VDOT to improve medication adherence for TB treatment. The aims are straightforward: 1) identify potential barriers and adapt the VDOT platform to add social bundle incentives transfer (qualitative); 2) pilot the DOT Selfie with incentives and evaluate treatment adherence; 3) build sustainable capacity for m-Health research in Uganda. The proposal builds on the qualitative analysis with appropriate focus groups and in-depth interviews, piloting in a RCT of 60 patients with newly diagnosed TB (DOT selfie vs DOT).
- The approach builds upon solid existing relationships including providers, software developers and technical support.
- The sample size justification is rational, in that they honestly don't try to show a significant difference in outcomes because of the small sample size, but they will be able to evaluate trends over time in adherence which is a reasonable start for this evaluation.
- The 3rd aim is the train 6 RAs and 4 National TB program RNs on the general principles of m-Health and the study protocol.

Weaknesses

- The sample size is a concern.

5. Environment:

Strengths

- Excellent research environment at UGA and Makerere.

Weaknesses

- None.

Study Timeline:

Strengths

- Reasonable pilot study of VDOT vs DOT in an area that needs an alternative to DOT. The rational for a RCT is strong.

- Timeline is reasonable, patient population should be enough, a backup plan is in place if enrollment is low.

Weaknesses

- Pilot nature a minor concern.

Protections for Human Subjects:

Acceptable Risks and/or Adequate Protections

- Strong protection described.

Data and Safety Monitoring Plan (Applicable for Clinical Trials Only):

Acceptable.

- o Actually, quite low risk but plans in place to monitor.

Inclusion of Women, Minorities and Children:

- Sex/Gender: Distribution justified scientifically
- Race/Ethnicity: Distribution justified scientifically
- For NIH-Defined Phase III trials, Plans for valid design and analysis: Scientifically acceptable
- Inclusion/Exclusion of Children under 18: Excluding ages <18; justified scientifically

- Appropriate in that adults more likely to have smartphones and to be able to follow the messages.

Vertebrate Animals:

Not Applicable (No Vertebrate Animals)

Biohazards:

Not Applicable (No Biohazards)

Applications from Foreign Organizations:

Justified

Select Agents:

Not Applicable (No Select Agents)

Resource Sharing Plans:

Acceptable

- Appropriate.

Authentication of Key Biological and/or Chemical Resources:

Not Applicable (No Relevant Resources)

Budget and Period of Support:

Recommend as Requested

CRITIQUE 6

Significance: 1
Investigator(s): 2
Innovation: 2
Approach: 2
Environment: 1

Overall Impact: This application proposes to pilot a mobile phone-based video selfie app (VDOT) to monitor pill intake to improve drug adherence among patients receiving TB treatment in Uganda. This is a highly innovative application, which can potentially make a substantial impact on TB treatment adherence and ultimately improve TB treatment outcomes in resource-limited settings. The scientific premise of this application appears to lie in these aspects: 1) treatment adherence is key to the success of TB treatment, 2) the proposed VDOT app offers a low-cost, effective adherence monitoring tool to improve TB adherence. Thus, the overall public health impact of this application is deemed to be on that, if the proposed VDOT is proven to be effective, it will provide a valuable mHealth tool in treatment adherence, which may go far beyond TB control.

1. Significance:

Strengths

- TB is deadly and a serious global health issue. TB control via effective TB treatment is critical to both public health and individual patient's health welfare. The proposed the VDOT has the potential to improve patient's TB treatment adherence. Understanding and measuring its actual effectiveness via gold standard randomized clinical trials will help with the clinical practice to actually adopt the technology in the TB control.

Weaknesses

- No major weakness identified.

2. Investigator(s):

Strengths

- The PI, Dr. Sekandi, is well trained in TB epidemiology and mHealth research. She has assembled an impressive investigative team with complementary experiences. She is well qualified to lead the team.

Weaknesses

- No major weakness identified.

3. Innovation:

Strengths

- This is a highly innovative application to use video-based VDOT in monitoring TB treatment adherence.

Weaknesses

- No major weakness identified

4. Approach:

Strengths

- The proposed pilot randomized clinical trial to test the effectiveness of VDOT in adherence monitoring is proper. Trial parameters are well-thought and justified. Statistical considerations are well presented. Plans are in place to account for study limitations.

Weaknesses

- No major weakness identified.

5. Environment:

Strengths

- The research sites at UG and Makerere SPH are excellent.

Weaknesses

- No major weakness identified.

Study Timeline:

Strengths

- Study timeline is presented and considered comprehensive and justified.

Weaknesses

- More time shall be given to the IRB approval given both US and Uganda sites are involved.

Protections for Human Subjects:

Acceptable Risks and/or Adequate Protections

- The proposed plan for human subject's protection is comprehensive and justified.

Data and Safety Monitoring Plan (Applicable for Clinical Trials Only):

Acceptable

  - The proposed DSM plan is comprehensive with interim analysis planned for trial monitoring.

Inclusion of Women, Minorities and Children:

- Sex/Gender: Distribution justified scientifically
- Race/Ethnicity: Distribution justified scientifically
- For NIH-Defined Phase III trials, Plans for valid design and analysis: Not applicable
- Inclusion/Exclusion of Children under 18: Excluding ages <18; justified scientifically
- The inclusion of women and minorities, and the exclusion of children under age 18 are justified.

Vertebrate Animals:

Not Applicable (No Vertebrate Animals)

Biohazards:

Not Applicable (No Biohazards)

Applications from Foreign Organizations:

Justified

- Given the proposed DOT Selfie will be tested for TB control in Uganda, the inclusion of Makerere University is appropriate and justified.

Select Agents:

Not Applicable (No Select Agents)

Resource Sharing Plans:

Unacceptable

- Although a study dissemination plan is presented, it may not replace a more detailed comprehensive resource sharing plan.

Authentication of Key Biological and/or Chemical Resources:

Not Applicable (No Relevant Resources)

Budget and Period of Support:

Recommend as Requested

THE FOLLOWING SECTIONS WERE PREPARED BY THE SCIENTIFIC REVIEW OFFICER TO SUMMARIZE THE OUTCOME OF DISCUSSIONS OF THE REVIEW COMMITTEE, OR REVIEWERS' WRITTEN CRITIQUES, ON THE FOLLOWING ISSUES:

PROTECTION OF HUMAN SUBJECTS: ACCEPTABLE

INCLUSION OF WOMEN PLAN: ACCEPTABLE

INCLUSION OF MINORITIES PLAN: ACCEPTABLE

INCLUSION OF CHILDREN PLAN: ACCEPTABLE

COMMITTEE BUDGET RECOMMENDATIONS: The budget was recommended as requested.

Footnotes for 1 R21 TW011365-01; PI Name: Sekandi, Juliet Nabbuye

Ad hoc or special section application percentiled against "Total CSR" base.

NIH has modified its policy regarding the receipt of resubmissions (amended applications). See Guide Notice NOT-OD-14-074 at http://grants.nih.gov/grants/guide/notice-files/NOT-OD-14-074.html. The impact/priority score is calculated after discussion of an application by averaging the overall scores (1-9) given by all voting reviewers on the committee and multiplying by 10. The criterion scores are submitted prior to the meeting by the individual

reviewers assigned to an application, and are not discussed specifically at the review meeting or calculated into the overall impact score. Some applications also receive a percentile ranking. For details on the review process, see http://grants.nih.gov/grants/peer_review_process.htm#scoring.
